# Selective manipulation of the inositol metabolic pathway for induction of salt-tolerance in *indica* rice variety

**DOI:** 10.1038/s41598-019-41809-7

**Published:** 2019-03-29

**Authors:** Rajeswari Mukherjee, Abhishek Mukherjee, Subhendu Bandyopadhyay, Sritama Mukherjee, Sonali Sengupta, Sudipta Ray, Arun Lahiri Majumder

**Affiliations:** 1 Division of Plant Biology, Bose Institute, Kolkata, India; 2 Department of Botany, Bethune College, Kolkata, India; 3 School of Plant Environment and Soil Sciences, Lousiana State University Agricultural Center, Lousiana, USA; 4 Department of Botany, Centre of Advanced Studies, University of Calcutta, Kolkata, India

**Keywords:** Molecular engineering in plants, Abiotic

## Abstract

Halophytes are rich sources of salt stress tolerance genes which have often been utilized for introduction of salt-tolerance character in salt-sensitive plants. In the present study, we overexpressed *PcINO1* and *PcIMT1* gene(s), earlier characterized in this laboratory from wild halophytic rice *Porteresia coarctata*, into IR64 *indica* rice either singly or in combination and assessed their role in conferring salt-tolerance. Homozygous T_3_/T_4_ transgenic plants revealed that *PcINO1* transformed transgenic rice lines exhibit significantly higher tolerance upto 200 mM or higher salt concentration with negligible compromise in their growth or other physiological parameters compared to the untransformed system grown without stress. The PcIMT1-lines or the double transgenic lines (DC1) having *PcINO1* and *PcIMT1* introgressed together, were less efficient in such respect. Comparison of inositol and/or pinitol pool in three types of transgenic plants suggests that plants whose inositol production remains uninterrupted under stress by the functional PcINO1 protein, showed normal growth as in the wild-type plants without stress. It is conceivable that inositol itself acts as a stress-ameliorator and/or as a switch for a number of other pathways important for imparting salt-tolerance. Such selective manipulation of the inositol metabolic pathway may be one of the ways to combat salt stress in plants.

## Introduction

Abiotic stress inflicted on the sessile plants implies ~65% loss of yield for crop plants each year^1–3^ where salinity stress itself is a major contributor^4,5^. Hence, modified value-added crop plants with higher productivity potential under adverse conditions are of utmost importance for agriculture.

Cereals like rice (*Oryza sativa* L.) are well-known cost-effective source of calories^6^, rice being the staple food for millions of people^7^. Lamentably, there still remains a sizable gap in productivity to cope with escalating World’s population. Although conventional breeding programmes have resulted in development of some salt and drought-tolerant rice varieties and several lines have been released in the Philippines, Bangladesh and India^8^, the success rate of conventional breeding is not ample. Transformation of rice through genetic manipulation thus becomes a better option for stress management.

Studies with eukaryotes demonstrated, that inositol-based cytosolic solutes can function as protective compounds under stressed conditions. Thus, inositol became a critical component in biological systems since its first isolation. Inositols are essential for growth in many yeast, fungi, plants and animals^9^ and its most abundant isoform, *myo*-inositol, occupies a central position in cellular metabolism^10,11^. Depletion of inositol in the fungal, plant or animal system induces cell death termed as “inositol-less death”^12^. Inositol and its metabolic intermediates like inositol polyphosphates (InsPs), galactinol, raffinose-family oligosaccharides (RFOs), methylated derivatives like pinitol, cell wall polysaccharides and phosphoinositides also participate in the crucial biological processes such as signal transduction^13,14^, membrane trafficking^13^, mRNA export^15^, stress tolerance^16,17^ and phosphorus storage^18,19^. In addition, the primary breakdown product of inositol, d-glucuronic acid, is utilized in the synthesis of various cell wall pectic and non-cellulosic compounds and ascorbic acid^10,20,21^. Methylated derivatives of inositol have been found to be effective in stalking ROS and thereby protecting photosynthetic machinery^22^.

A number of metabolic pathways have earlier been manipulated for production of essential metabolites along with overexpression of regulatory genes to confer salt-tolerance to different plants^23^. In the present work, we attempted manipulation of the inositol metabolism which involves a major biochemical network in transgenic rice lines by overexpressing two genes from *Porteresia coarctata viz*. the salt-tolerant *myo*-inositol phosphate synthase (MIPS) coding gene (*PcINO1*)^24^ producing inositol and the inositol methyl transferase gene (*PcIMT1*)^17^ producing pinitol *in planta*. All the transgenics were studied in terms of their growth, development and other characteristics under salinity stress.

## Results

### Test of allergenicity for candidate protein(s)

Rice being an edible crop, before introducing any external gene(s) into rice genome, cross testing for allergenicity of the protein products of the candidate gene(s) was thought to be prudent. Protein sequences of PcIMT1 and PcINO1 were analyzed through *AllergenOnline Database v15 (January 12, 2015)*. Query sequence of PcIMT1 protein exhibited 110 amino acids match with an allergen *Solenopsis invicta* of 346 amino acids (protein ID: gi 51093373) and the second match was of 137 amino acids with an allergen *Cupressus sempervire* of 367 amino acids (Protein ID: gi 8101715). Both alignments of PcIMT1 with two allergens indicate a match of only 25.5% (*E*-value-0.14; Supplementary Fig. S1) and 24.8% (*E*-value-0.78; Supplementary Fig. S2) respectively, values indicative of non-allergenicity of the protein. The query sequence of PcINO1 protein exhibited no allergenicity when searched throughout the same database.

### Identification of embaryogenic stage in IR-64 calli through SEM imaging

Prior to transformation, SEM study were conducted taking ~10 days, ~20 days and ~30 days old IR-64 calli for identification of proper embryogenic state. Texture of ~10 days old calli (Supplementary Fig. S3a–e) was very tight, mostly without prominent groove, with no specific structure/shape (Supplementary Fig. S3d). The creamish ~20 days old calli were with prominent heart, torpedo and globular shaped embryos along with protrusions of non-uniform shape and size (Supplementary Fig. S3f–m) and were thought to be ideal for effective transformation. ~30 days old calli (Supplementary Fig. S3n–p) showed numerous trichomes with dead tissues and with no heart/globular/torpedo shaped embryogenic structures.

### Construction of expression vectors with *PcINO1* and *PcIMT1* genes and generation of salt-tolerant transgenic rice lines

**Figure 1:**
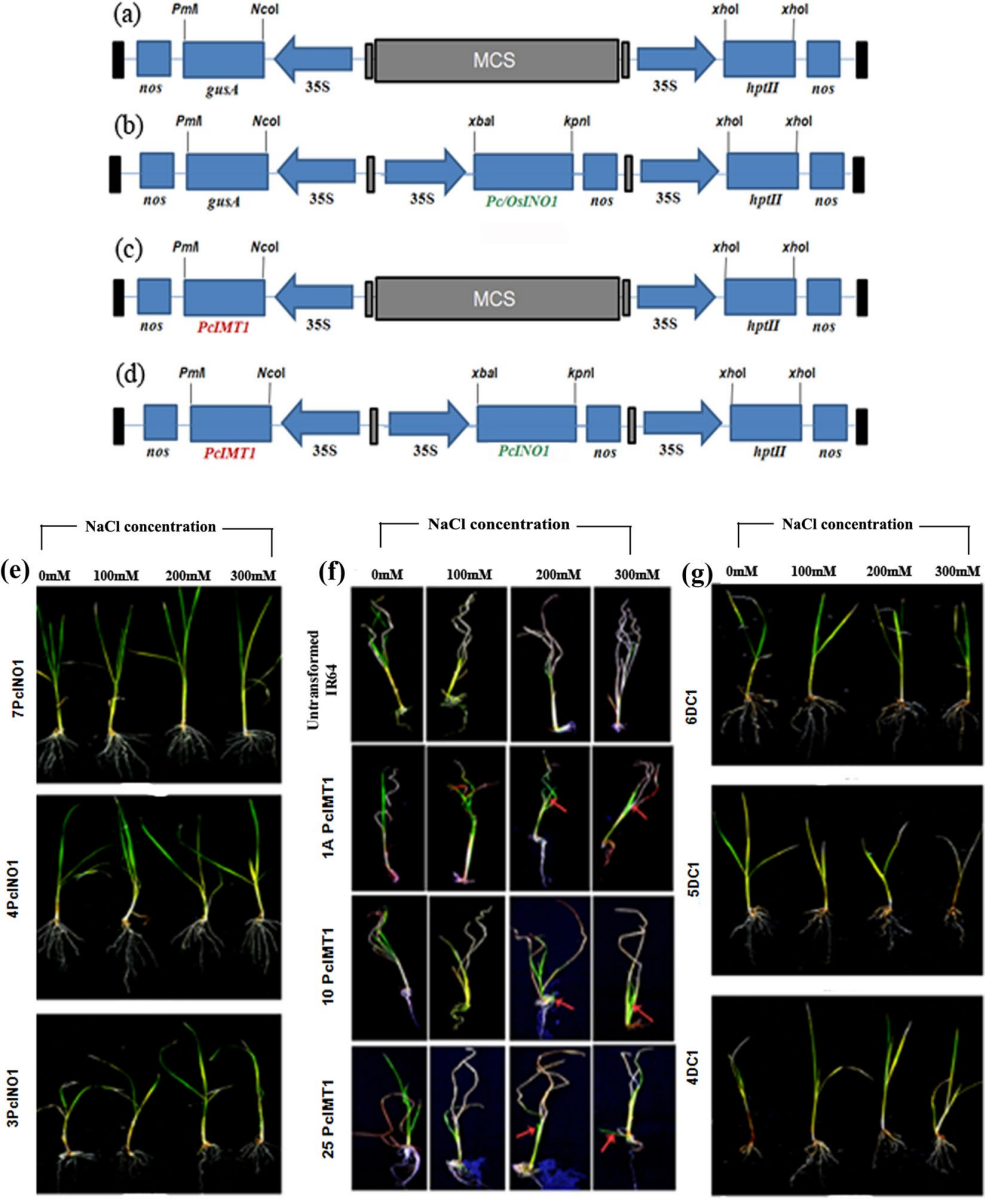
Representative pictures explaining vector-designs for plant-transformation and physiological response of selected transgenic lines under different NaCl concentrations kept for 7 days. (**a**) pCAMBIA1301 vector with no candidate gene at MCS site. (**b**) pCAMBIA1301*-PcINO1/OsINO1* construct containing *PcINO1/OsINO1* gene. (**c**) pCAMBIA1301*-PcIMT1* constructs containing *PcIMT1* gene in replacement of *gusA* gene. (**d**) pCAMBIA-*PcIMT1* + *PcINO1* Double Construct (DC1) containing both *PcIMT1* and *PcINO1* genes, each under individual *CAMV35S* promoters. (**e**–**g**) Respectively represents *PcINO*1, *PcIMT*1 and DC1 (*PcINO*1 + *PcIMT*1) gene-transformed transgenic lines.

Two candidate genes *viz. PcINO1* and *PcIMT1*, earlier isolated from *Porteresia coarctata*^17,24^ were cloned into pCAMBIA1301 (Fig. 1a) under *CAMV35S* promoter to generate pCAMBIA1301-*PcINO1/OsINO1* (Fig. 1b) and pCAMBIA1301-*PcIMT1* (Fig. 1c) construct. A single pCAMBIA1301 construct (termed DC1) was also generated where both *PcIMT1* and *PcINO1*genes were put together under individual *CAMV35S* promoters (Fig. 1d).

~20 days old embryogenic calli were transformed by adopting a modified *Agrobacterium-*mediated rice transformation protocol (Supplementary Fig. S4) following previously reported methods^25,26^. Among the T_0_ putative transgenics (Supplementary Fig. S5g–m) *hptII* gene specific PCR positive plants were selected and advanced to next generation. Homozygous T_2_ lines showing the presence of *hptII* gene at 1 kb, *PcINO1* gene at 1.5 kb and *PcIMT1* gene at 1.1 kb region respectively (Supplementary Fig. S5g–m) were selected for Southern blot analysis of plants from PcINO1-lines (110 plants), PcIMT1-lines (153 plants) and (*PcINO1* + *PcIMT1*) *i.e* DC1 (98 plants) lines. *PcINO1* gene probe highlighted two copies of endogenous *INO1* genes in all plants. Southern positive T_3_ PcINO1-plants exhibiting additional single-copy integration in another locus with specific *INO1* probe (Supplementary Fig. S6a–d) were used for segregation analysis (Supplementary Tables S4a–S6a). All the single copied transgenic plants (35 PcINO1-lines, 50 PcIMT1-lines, and 25 DC1-lines) were chosen for further analysis.

### Analysis of homozygous transgenic lines

T_3_ plants were grown under salt-stress of different concentrations (0 mM, 100 mM, 200 mM and 300 mM) for 7 days. Three best performing lines from each type of transgenic systems [7PcINO1, 4PcINO1 and 3PcINO1-lines (Fig. 1e); 25PcIMT1, 10PcIMT1 and 1*A*PcIMT1-lines (Fig. 1f); 6DC1, 5DC1, 4DC1-lines (Fig. 1g)] were selected for checking stress sustenance. Selected lines remained healthy and exhibited unfolding of new leaves under (200 mM–300 mM) salt-stress even after 7 days while rest of the lines showed noticeable dechlorophyllisation under 300 mM stress

**Figure 2:**
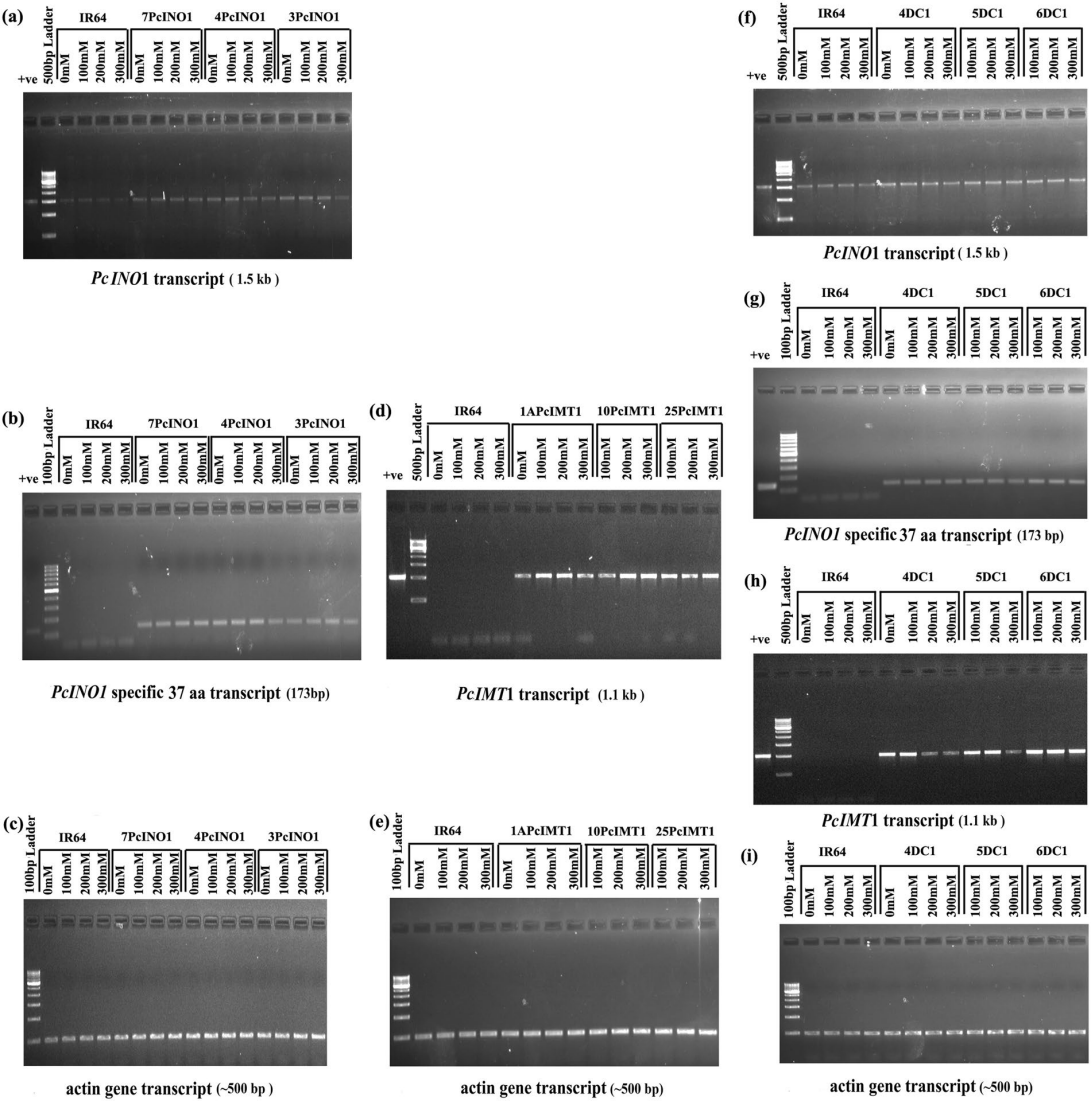
Transcript analysis of *PcINO1* & *PcIMT1* genes in three types of transgenic lines grown under varying NaCl concentrations for 7 days. (**a**–**c**) Represent *PcINO1* transcripts, *PcINO1* specific 37 amino acid transcripts and *Actin* gene transcripts (endogenous control) respectively in PcINO1:transgenic lines; (**d**,**e**) represent *PcIMT1* transcripts and *Actin* gene transcripts (endogenous control) respectively in PcIMT1:transgenic lines; (**f**–**i**) represent *PcINO1* transcripts, *PcINO1* specific 37 amino acid transcripts, *PcIMT1* transcripts and *Actin* gene transcripts (endogenous control) respectively in (PcINO1 + PcIMT1):double transgenics(DC1).

Transcript analysis of selected nine lines after 7 days of stress under different NaCl-concentrations (0 mM, 100 mM, 200 mM, 300 mM) shows the *INO1* transcript in the untransformed IR-64 and all three PcINO1-transgenic lines (Fig. 2a). 37-amino acid specific *PcINO1-*transcript^24^, specific to *PcINO1* gene was present in varying proportion in PcINO1-transgenic lines while being absent in the untransformed lines (Fig. 2b). *PcIMT1-*transcripts were found to vary its level with increase in salt concentration in PcIMT1-transgenic lines (Fig. 2d). The 6DC1 line exhibited highest level expression of *PcIMT1-*transcript at 300 mM salt in comparison to the other two DC1-lines (Fig. 2h).

**Figure 3:**
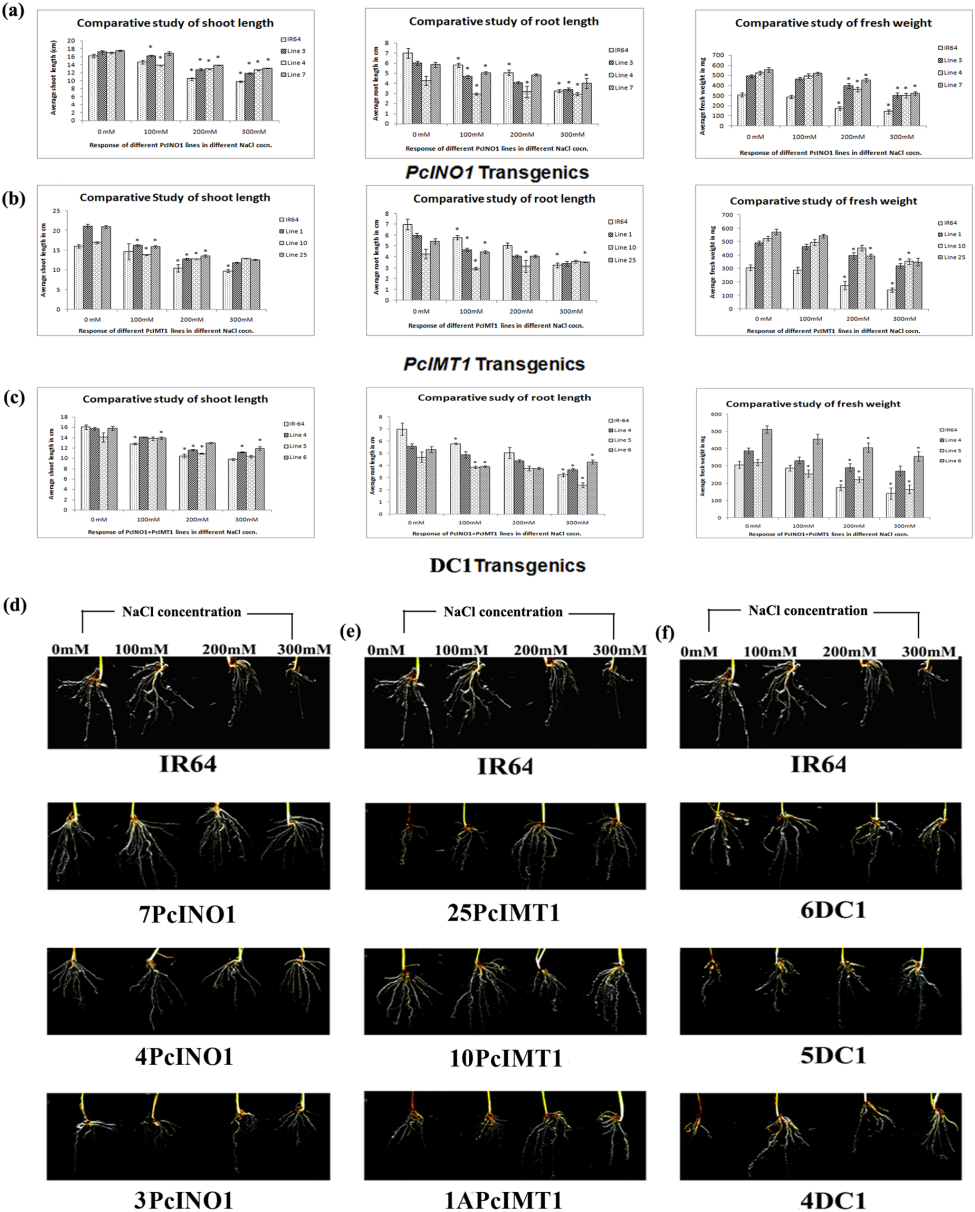
Comparative phenotypic study for shoot-length, root-length and fresh-weight of transgenics with untransformed lines under variable NaCl stresses kept for 7 days, followed by response of roots in transgenic lines. Bar diagrams exhibiting variation in phenotypic responses in three of each (**a**) PcINO1; (**b**) PcIMT1 and (**c**) DC1 (*PcINO1* + *PcIMT1*) transgenic lines under variable salt-stresses. Data represented average of three plants ± SD (P ≤ 0.05). (**d**–**f**) Representative root-responses of selected PcINO1, PcIMT1 and DC1 (*PcINO1* + *PcIMT1*) transgenic lines under stressed and unstressed condition.

**Figure 4:**
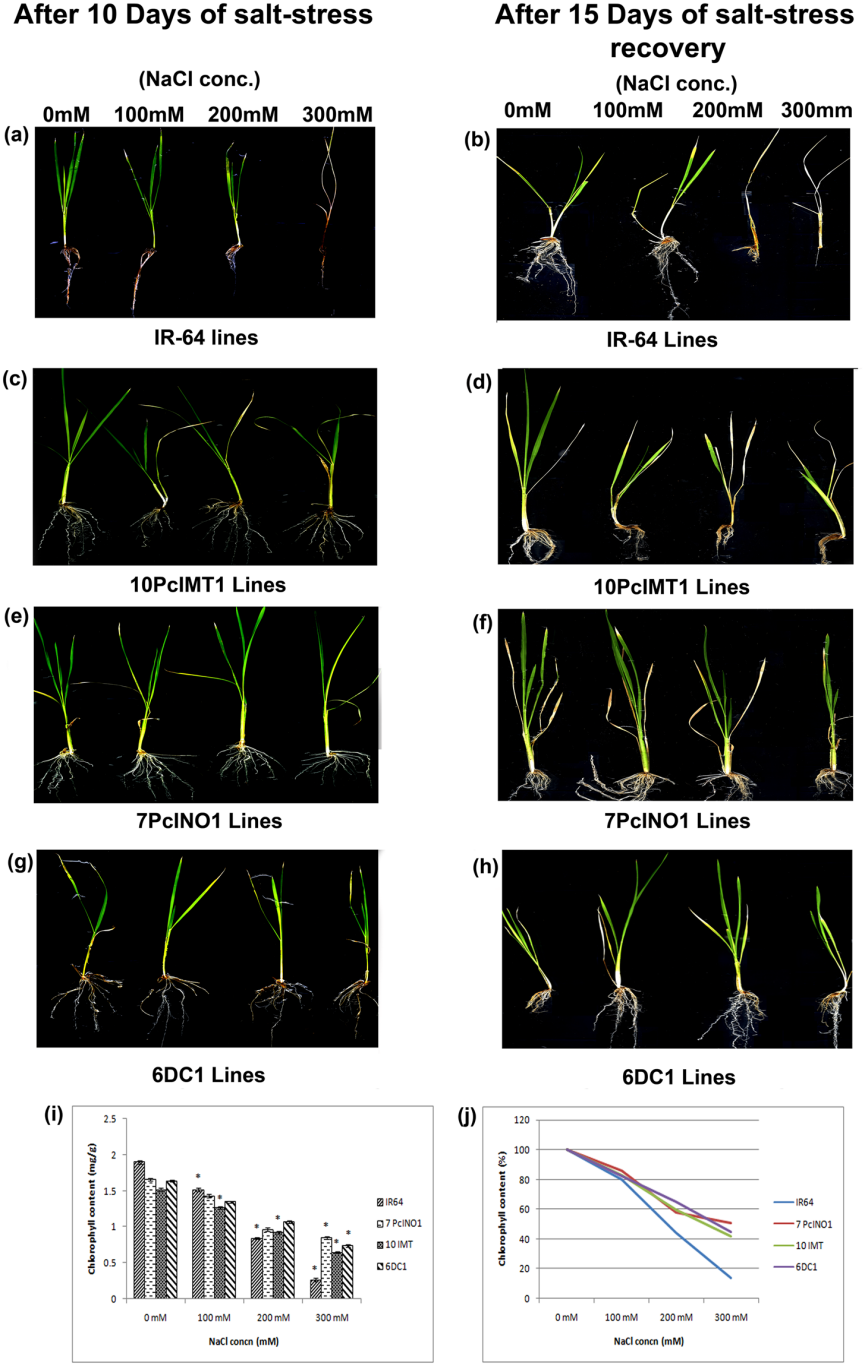
Comparative physiological response in terms of stress recovery and capacity of chlorophyll retention in transgenics and untransformed lines after withdrawal of differential NaCl stresses. (**a**) Untransformed plants under stress for 10 days and (**b**) after recovery of 15 days from stress. (**c**) 10PcIMT1 plants under stress for 10 days and (**d**) after recovery of 15 days from stress. (**e**) 7PcINO1 plants under stress for 10 days and (**f**) after recovery of 15 days from stress. (**g**) 6DC1 plants under stress for 10 days and (**h**) after recovery of 15 days of from stress. (**i**,**j**) Chlorophyll retention capacity of transgenic plants along with untransformed IR-64 lines under different salt stresses. Data represented the average of three plants ± SD (P ≤ 0.05).

Comparative shoot-length, root-length and fresh wt. taken after 10 days-stress indicated 7PcINO1-line, 10PcIMT1-line and 6DC1-line as the best in terms of growth parameters (Fig. 3a–c). Root growth under 200 mM/300 mM salt concentration was maximum in 7PcINO1-line, 10PcIMT1-line and 6DC1-line in comparison to other respective lines (Fig. 3d–f). Analysis through DIRT imaging software revealed generation of more lateral and adventitious roots, indicative of better stress tolerance (Fig. 3d–f and Supplementary Table S3). These three plant lines recovered best after 15 days of stress withdrawal considering growth and survivability (Fig. 4a–h). Even after 300 mM salt stress for 10 days, 7PcINO1-line, 10PcIMT1-line and 6DC1-line fully recovered their normal physiological activity. Chlorophyll estimation after 15 days of salt withdrawal prominently indicated recovery of chlorophyll content in all transgenic lines while untransformed IR-64 exhibited remarkable decline in chlorophyll content (Fig. 4i,j). However, it is evident, among the three types of transgenic lines, 7PcINO1-line showed best retention of chlorophyll content at 300 mM NaCl-stress (Fig. 4i,j).

Statistical analysis was finally made on T_6_ (PcINO1-lines) and T_4_ seeds (PcIMT1-lines & DC1-lines) of selected 9 lines on the basis of germination percentage (i.e. 95–100%) on media containing hygromycin (50 mg/L). Three transgenic lines were found to exhibit 100% germination frequency along with better performance under salt stress (Supplementary Table S4b:*PcINO1*-lines; Supplementary Table S5b:PcIMT1-lines; Supplementary Table S6b:DC1-lines). These lines were finally selected for determination of site of introgression by TAIL-PCR technique^27^. *PcINO1* transgene showed stable introgression into chromosome-8 while *PcIMT1* either singly or in combination with *PcINO1*, introgressed into chromosome-5 (Supplementary Table S1) in the homozygous transgenic IR-64 rice lines.

**Figure 5:**
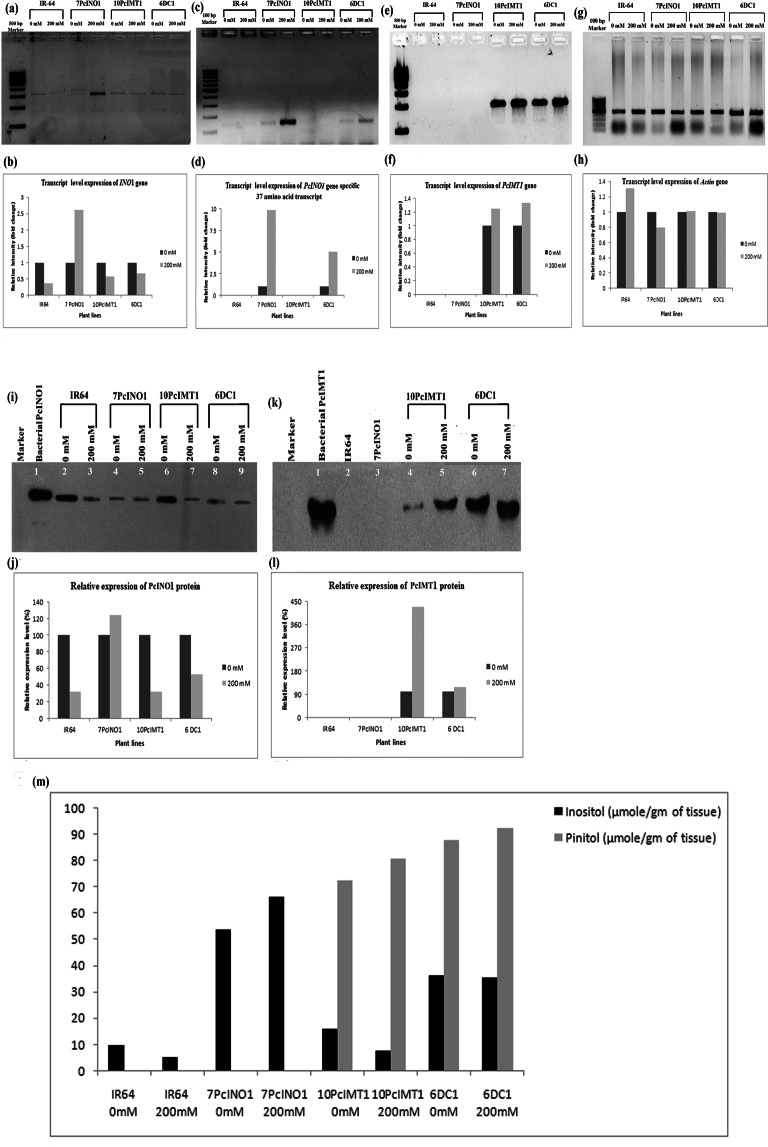
Transcript and Western blot analysis followed by estimation of metabolites in three transgenic lines kept under 0 mM & 200 mM NaCl stresses for 12 hrs. (**a**,**b**) RT-PCR exhibiting differential expression of endogenous *PcINO1* full-length transcript (1.5 kb) in transgenics and untransformed lines. (**c**,**d**) Exhibiting varying expression of 37-amino acid specific *PcINO1* gene-transcript (111 bp) in 7PcINO1 & 6DC1-transgenic lines. No expression found in 10PcIMT1 and untransformed line. (**e**,**f**) Exhibiting varying *PcIMT1* gene-transcript (1.1 kb) expression in 10PcIMT1 & 6DC1 transgenics. (**g**,**h**) Exhibiting full length *Actin* gene transcript (500 bp) in all lines as endogenous control. Data represented average of three replica sets ± SD (P ≤ 0.05). (**i**) Representative Western Blot charged with PcINO1 (62 kD) antibody; Lane1: Bacterial protein as positive control; Lane 2: untransformed IR-64 in 0 mM salt stress, Lane 3: untransformed IR-64 in 200 mM; Lane 4: 7PcINO1 transgenic in 0 mM salt stress; Lane 5: 7PcINO1 200 mM salt stress; Lane 6: 10PcIMT1 transgenic in 0 mM salt stress, Lane 7: 10PcIMT1 transgenic in 200 mM salt stress; Lane 8: 6DC1 transgenic in 0 mM salt stress, Lane 9: 6DC1 transgenic in 200 mM salt stress. (**j**) Graphical representation of PcINO1 protein in different transgenic lines considering, expression at 0 mM salt as 100%. (**k**) Blot charged with IMT1 (42 kD) antibody; Lane1: Bacterial protein as positive control; Lane 2: untransformed IR-64 lines in 0 mM salt stress; Lane 3: 7PcINO1 transgenic in 0 mM salt stress; Lane 4: 10PcIMT1 transgenic in 0 mM salt stress, Lane 5: 10PcIMT1 transgenic in 200 mM salt stress; Lane 6: 6DC1 transgenic in 0 mM salt stress, Lane 7: 6DC1 transgenic in 200 mM salt stress. Prestained (10–180) KD protein ladder markers were used in both the gels. (**l**) Graphical representation of PcIMT1 protein in different transgenic lines, considering expression at 0 mM salt as 100%. Data represented average of three replica sets ± SD (P ≤ 0.05). (**m**) Graphical representation of comparative estimation of metabolites in transgenics through GCMS from different transgenic plants in absence (0 mM) and presence (200 mM) of salt stress for 12 hrs. Data represented average of three replica sets ± SD (P ≤ 0.05).

Selected T_4_ plant lines from each type of transgenic systems *viz*. 7PcINO1, 10PcIMT1 and 6DC1 were subjected to 12 hrs salt stress (0 mM & 200 mM) to compare the transcript with their corresponding protein level expression for each transgene. Figure 5a–h represented all transcripts in individual gel slots. In Fig. 5a, accumulated *INO1* transcript of *OsINO1*/*PcINO1* was detected in all plants along with IR-64 untransformed line. In comparison to the untransformed control line, 7PcINO1-line recorded a ~2.61 fold increase in the *INO1* transcript under stress as against ~0.5 fold in 10PcIMT1 line and 0.67 fold in 6DC1 line under similar conditions (Fig. 5a,b). *PcINO1* specific 37-amino acid gene transcript level expression was maximum in 7PcINO1-lines (~10-fold higher than in 0 mM) over 6DC1 line (~5-fold higher than in 0 mM) (Fig. 5d) while it was absent in 10PcIMT1-line. In case of *PcIMT1-*transcript an increase of ~1.3- to ~1.4-fold for both 10PcIMT1 and 6DC1-line has been found (Fig. 5f).

Western blot analysis revealed that under stress PcINO1 protein was maximally expressed in 7PcINO1-line compared to the IR-64 or the 10PcIMT1 or the 6DC1 lines (5i,j). Accumulation of PcIMT1 protein was detected in both PcIMT1/DC1-lines, albeit, not detectable in untransformed IR-64 or the PcINO1-lines (5k,l). Identification and estimation of the metabolite products of expressed transgene(s) *viz*. inositol and pinitol through GC-MS analysis (Fig. 5m) validated the transcript and the western blot data obtained. The metabolite accumulation was found to be in line with the trend of transcript and protein expression. Decrease in inositol content was observed under salt stress in almost similar manner in untransformed IR-64 and 10PcIMT1-lines whereas 6DC1 (*PcINO1 + PcIMT1*) plants exhibited almost no change in inositol pool under salt-stress compared to no-salt condition. However, 7PcINO1-lines maintained an increasing trend of inositol production even under salt-stress. Pinitol, detected only in 10PcIMT1 and 6DC1-lines, was found to increase in 10PcIMT1-line, whereas 6DC1-line showed similar increase under 200 mM salt-stress compared to no-salt condition (Fig. 5m).

### Detection and study of photosynthetic ability of the transgenic plants

**Figure 6:**
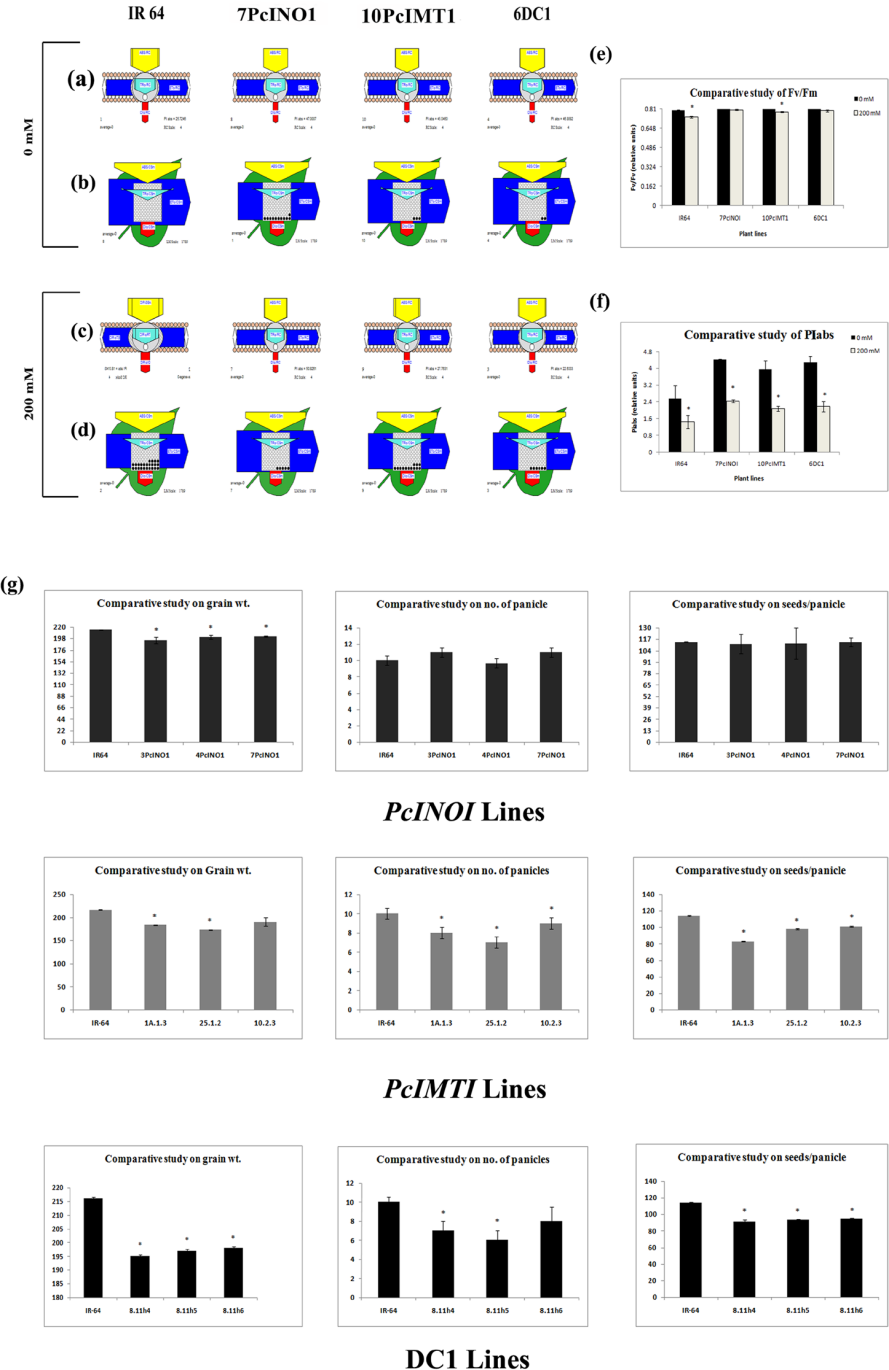
Representative pictures showing light entrapping capacity of transgenics and untransformed IR-64 lines during photosynthesis under salt stresses (0 & 200 mM NaCl) kept for 12 hrs. Agronomic characters in transgenic and untransformed lines have been studied. (**a**,**b**) Specific (membrane) model displaying specific fluxes per PSII reaction centre under 0 mM NaCl stress in untransformed and transgenic plants. [Yellow pentagon, absorption maxima per reaction center; Green pentagon, specific flux for trapping per reaction center and red pipe, specific flux for dissipation per reaction center]. (**c**,**d**) Phenomenological yield models per excited cross-section under 200 mM NaCl stress in untransformed and transgenic plants [Yellow triangle, absorption maxima per excited cross-section (ABS/CSm); blue pipe, electron transport rate per excited cross-section (ET0/CSm); green triangle, trapped energy per excited cross-section (TR0/CSm); red block, dissipation maxima per excited cross-section (DI0/CSm); empty circles, active reaction centers; filled circles, inactive reaction centers.] (**e**) Photosynthetic efficiency (in terms of maximum quantum yield of photosystem-II; Fv/Fm) measurement (**f**) Over all photosynthetic performance index, PI[abs] measurement in untransformed and transgenic plants under 0 and 200 mM NaCl. (**g**) Comparative study of agronomic characters (grain weight, no. of panicles & no. of seeds) for untransformed and transgenic plants under normal growth conditions. Data represented the average of three plants ± SD (P ≤ 0.05).

7PcINO1/10PcIMT1/6DC1-transgenic lines along with untransformed line were kept under 0 mM and 200 mM NaCl-stress for 12 hrs to check for any change in their photosynthetic-capacity. Figure 6a,b represent no blockage in photo-centre during photosynthesis for untransformed IR-64 under no-salt condition while 7PcINO1, 10PcIMT1 and 6DC1-line recorded insignificant blockage. After 12 hrs of 200 mM salt-stress, IR-64 plant exhibited considerable blockage, while rest of the transgenic systems showed negligible blockage at the photo-centre (Fig. 6c,d). Further analysis showed almost unaffected photosynthetic functions in terms of Fv/Fm ratio for each set (Fig. 6e) and the absolute photosynthetic values (PIabs) as plotted in Fig. 6f. PIabs values for the transgenic systems were higher than the untransformed system.

### Agronomic characters of the transgenic plant lines

**Figure 7:**
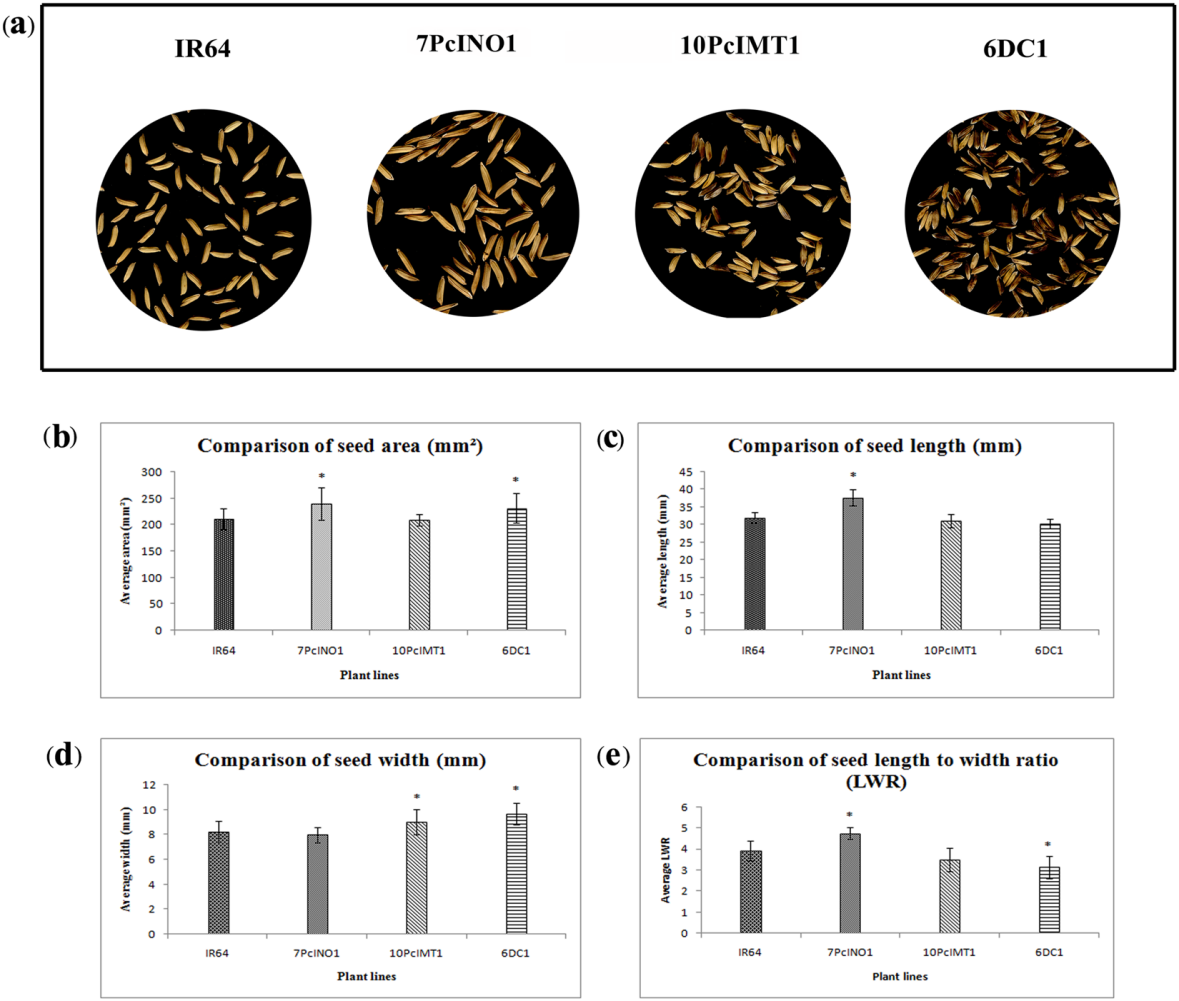
Morphological analysis of seed characters of different transgenics and untransformed lines. (**a**) Representative seed profiles analysed by SMART-GRAIN software. (**b**) Graphical representation of comparative study among seed area (mm^2^), (**c**) seed length (mm), (**d**) seed width (mm) and (**e**) seed length to seed width ratio (LWR) of different transgenics and untransformed line. Data represented the average of 100 seeds per plant line ± SD (P ≤ 0.05).

T_3_ transgenic plants from 7PcINO1, 10PcIMT1 and 6DC1 lines along with untransformed IR-64 at maturity were self-pollinated. After seed setting and maturation of the panicle, agronomic characters like grain wt., number of panicles and number of seeds per panicle were determined (Fig. 6g). Under normal condition, all these transgenic plants were able to produce matured seeds (Fig. 7a) and could maintain normal growth. Grain quality from transgenic plants, especially, of the 7PcINO1-line, as analyzed with SMART-GRAIN software was comparable or marginally better in length and width than the untransformed IR-64 control line (Fig. 7b–e).

### Generation of a potent transgenic IR-64 rice line over-expressing endogenous *OsINO1* gene

To compare *in planta* activity of *PcINO1* gene in PcINO1*-*transgenic rice lines in comparison with the overexpression of *OsINO1* gene, parallel attempts were made to develop IR-64 rice lines overexpressing endogenous *OsINO1* gene. 22 PCR positive plants were selected out of 5 transformation events (Supplementary Fig. S7a,b). Three T_3_ homozygous OsINO1-transgenic lines were selected through Southern blot analysis (Supplementary Fig. S7c). The germination percentage of the *PcINO1/OsINO1* introgressed and control seeds under increasing NaCl concentrations was monitored in different independent transgenic lines (Supplementary Fig. S7d). Increase in NaCl concentration caused pronounced germination inhibition in control and *OsINO1* transformed plants whilst *PcINO1* transformed plants showed, albeit, less vigorous germination upto 400 mM NaCl at least for 10 days (Supplementary Fig. S7e). Growth parameters like shoot and root length (data taken after 15 days of salt stress) were also found to be affected negatively in the presence of higher concentration of salt in control and *OsINO1* plants whereas most of the 7PcINO-plants showed better performance (Supplementary Fig. S7f,g). Photosynthetic performance parameters like chlorophyll-*a* fluorescence of control plants and PcINO1*/*OsINO- plants under saline environment, showed that PcINO1-plants are much better adapted to saline environment than the untransformed control and OsINO1-plants. These results indicate a substantial protection of photosystems, especially, PSII in PcINO1-transgenic plants (Supplementary Fig. S7h,i).

### Comparative Transcriptome analysis by cDNA microarray

**Figure 8:**
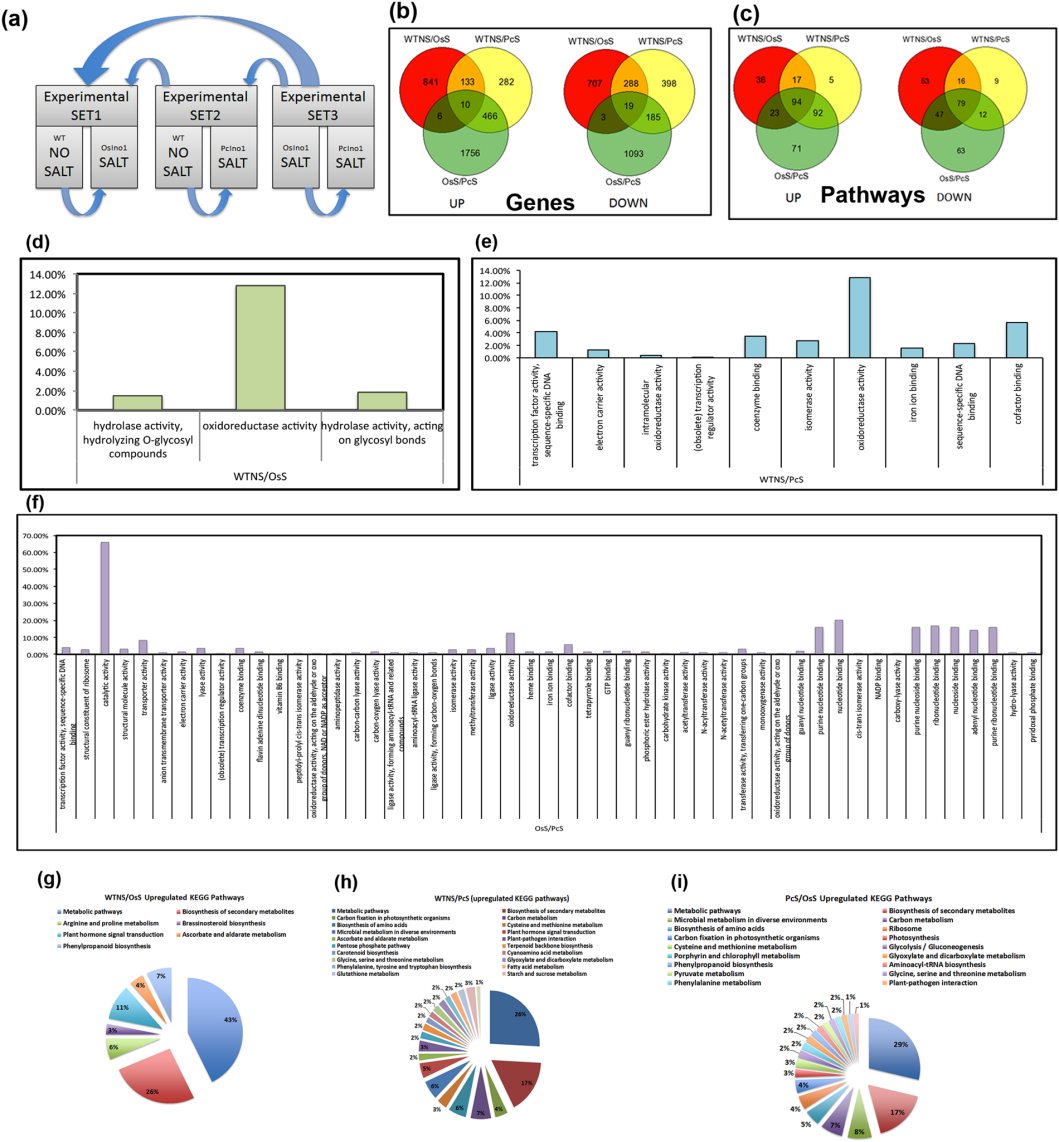
Comparative microarray data analysis for *PcINO1/OsINO1* transgenic plants and untransformed line. (**a**) Schematic representation of the experimental design. (**b**) Up-regulation and down-regulation of total gene-transcripts under salt and no salt condition. (**c**) Up-regulation and down-regulation of different pathways under salt and no salt condition. (**d**–**f**) Enrichment of GO terms are shown in comparative profile of the transgenics. (**g**–**i**) Comparative analysis of enrichments in the KEGG pathways.

To evaluate transactivation capacity of inositol overexpression in rice under stress, comparative transcriptome of inositol-overexpressor lines were attempted through a strategy outlined in Fig. 8a. Although inositol overproduction may elevate osmoprotectant status of the plant there are several other pathways that may participate in the process either as indirect response or as causality. A total of 1252 and 1420 transcripts were up and down-regulated respectively in OsINO1-transformed plants in salt compared to control condition. A total of 1493 and 1403 transcripts were up and down-regulated in PcINO1-transformed plants in salt. However, when differential transcripts from *PcINO1*-transformed plants were compared with OsINO1-transformed plants in salt, the total numbers of up-regulated transcripts rise to 3183 and down-regulated transcripts were 1607 in number (Fig. 8b). Interestingly, the Venn diagram for overlapping categories shows that on overexpression of *PcINO1*, salt up-regulates at least 1756 unique transcripts and down-regulates no less than 1093 unique transcripts compared to *OsINO1* overexpressor in salt (Fig. 8b). We analyzed the GO terms associated with up-regulated and down-regulated transcripts and enriched the significant terms using the rice genome as reference. The up-regulated genes were assigned to specific pathways according to KEGG database. 94 common pathways are up-regulated in all 3 comparative sets, whereas 71 pathways were uniquely enriched in *PcINO1*-transgenics in salt (Fig. 8c).

Enrichment of GO terms and KEGG pathways are shown in comparative profile of the transgenics (Fig. 8d–i). The major up-regulated KEGG pathways in PcINO1- transgenics compared to OsINO1-transgenics belong to metabolic pathways, biosynthesis of secondary metabolites, and photosynthetic carbon-fixation. The up-regulated GO terms in PcINO1-lines are majorly constituted by catalytic processes/enzymatic reactions; which signifies the presence of salt-resistant or salt-responsive enzymatic processes in PcINO1-transgenic lines which are coordinately expressed.

## Discussion

An analysis of the pathways for inositol utilization (Supplementary Fig. S8) would reveal the number of pathways emanating from inositol which are intimately connected to stress responses in plants. These include the methylation by IMT1 function to generate pinitol and other methylated inositols^28–30^, generation of RFOs like galactinol and the raffinose series of oligosaccharides through GolS^31,32^, cell-wall polysaccharides through the MIOX^33,34^ and the phosphoinositides^35^. Interestingly, all these metabolic events originate from a single evolutionarily conserved pathway namely the L-*myo*-insitol 1-phospahte synthase (MIPS) reaction, resulting in accumulation of inositol for conversion to other metabolites of interest^31^. Hence, generation of inositol through the MIPS reaction is of vital importance to the successful operation of the entire inositol metabolic cycle. In contrast to the other pathways for synthesis of osmoprotectants^23^ such as glycine-betaine, proline, trehalose etc, which are products of specialized extension of specific pathways, synthesis of inositol is carried out by an evolutionarily conserved single enzyme^36–38^. Under salt-environment, however, this pathway in most glycophytes is known to record decreased MIPS activity, thus affecting the channelization of inositol flow throughout the inositol metabolic cycle.

Since halophytic plants are known to be good source of genes and promoters of salt-tolerance^39^, our attention was initially drawn on the wild halophytic rice, *Porteresia coarctata*, wherein we looked for either increased or tolerant MIPS activity under salt-environment^40,41^. The subsequent discovery of an unique salt-tolerant MIPS coding *PcINO1* gene^24^, elucidation of the mechanism of its salt-tolerance property^11^ and demonstration of its capability to confer salt-tolerance to varied pro-and eukaryotic organisms including higher plants^42^, opened up the possibility of raising salt-tolerant crop plants by transgenic introgression of the *PcINO1* gene. Further, the co-introgression of *PcINO1* and *McIMT1*, from *Mesembryaumthemum crystallinum*^43,44^, enabled achieving a salt-tolerant phenotype in transgenic tobacco^22^. Such observations validated that likewise, the *PcINO1* and *PcIMT1* gene, isolated from *Porteresia coarctata*^17,24^, may improve salt tolerance in a crop genetic background. Based on such proof of concept, an attempt has been made to generate salt-tolerant *indica* rice plants through transgenic functional co-introgression of both *PcINO1* and *PcIMT1*.

Seven different types of calli are generally induced from mature rice seeds^45^, differing in morphological characteristics and potential for plant regeneration^46^. Hence, identification of proper explants *i.e* rice calli at exact embryogenic stage is required to improve rice transformation efficiency^47^. In the present study, SEM analysis revealed maximum number of heart and/globular shaped embryos were observed in 20–21 days old tightly bound IR-64 calli (Supplementary Fig. S3) which served as the basic explants for plant transformation experiments.

Following identification of the MIPS proteins as non-allergenic (Supplementary Figs S1 and S2), plant expression cassettes were designed for *PcIMT1, PcINO1* and (*PcIMT1* + *PcINO1*) gene(s) under *CAMV35S* promoter for rice transformation to generate single copy transgene integrated T_2_ lines as judged by Southern blot analysis (Supplementary Fig. S6). Three Southern positive transgenic lines from each type were finally selected based on their performance under salt-environment (Fig. 1e–g) as well as their transcript analysis of the respective gene(s) (Fig. 2). Expression of different gene(s) transcripts (Fig. 5b,d,f,h) matched well with corresponding protein accumulation in immunoblots (Fig. 5j,l) and the metabolites such as inositol and pinitol in respective samples (Fig. 5m). These data confirm functional expression of the gene(s) and their protein(s) exhibiting corresponding enzymatic activities in the transgenic system suggesting a direct correlation among them. Site specificity of the transgene integration may attribute to the prominent increase in CaMV35S promoter mediated *PcINO1* transcription.

Being the first organ to sense stress insult, root growth and architecture are good indicators for stress perception^48^. Three lines *viz*. 7PcINO1, 10PcIMT1and 6DC1 exhibited better root growth than the other lines under 300 mM NaCl stress. After 10 days of salt-stress, greater number of adventitious roots was observed to develop when analyzed through DIRT imaging software (Supplementary Table S3). *Sorghum* plants are known to develop adventitious roots capable of accumulation of sodium and chloride ions under stress with enhanced adventitious root imparting salt tolerance^49^. Stimulation of the lateral root formation in *Arabidopsis thaliana* under drought stress and increased growth of roots in depth was found in rice^50–52^. In our experiment, transgenics imparting better tolerance under salt stress were found to have longer primary tap root system growing deeper under the ground. They also exhibited positive geotropism under stress contrary to the earlier reports of negative geotropism^53,54^. While high NaCl levels inhibit lateral root formation, lower NaCl levels are known to stimulate lateral root formation in an auxin-dependent manner^51,55^. Our results show that there was no remarkable decrease in number of lateral roots under salt stress despite reports of conflicting results^48,51,56^. In our experiment, transgenics (T_3_) showed better average root density than the untransformed control (Fig. 3d–f). Selected transgenic lines (7PcINO1, 10PcIMT1, 6DC1) recovered from stress impact to normal growth by maintaining their physiological activity (Fig. 4a–h).

Stress generates enormous reactive oxygen species (ROS) having a negative impact on photosynthetic machinery and the total chlorophyll content of a plant^57^. Chlorophyll reduction under abiotic stress symbolizes osmotic/oxidative stress, which may result from pigment photo-oxidation and chlorophyll degradation^58,59^. However, ectopic expression of various genes (viz. Annexin, VTE1 and mtlD) helps in retention of greater chlorophyll content under dehydration stress^60^. Since photosynthesis is affected by leaf chlorophyll content^61^, chlorophyll retention capacity under salt stress was checked quantitatively. Transgenic 7PcINO1-plants retained the chlorophyll content thus maintaining normal photosynthetic potential even under high salt concentration (Fig. 4i) while untransformed line showed maximum chlorophyll depletion.

Salinity affects plant growth by inducing changes in photosynthesis^62^ indicating that prompt reaction of photosynthetic machinery is a key factor for combating such stress effects^63,64^. Transgenic plants under 200 mM NaCl stress showed less number of closed photoreaction centre and higher F_v_/F_m_ value than that of untransformed IR-64 plants indicating better photosynthetic capability, 7PcINO1-line being the best identified line (Fig. 6e,f).

Agronomic characters of transgenics in T_4_ generation and above were studied along with untransformed IR-64 line under green house conditions (Fig. 6g). Numbers of panicles with matured grains in T4 transgenics (Fig. 6g) were compromised to a smaller extent in case of PcIMT1-line and DC1-transgenic lines when compared with PcINO1-transgenic lines, keeping all other agronomic characters intact with better salt-tolerance. This fact is indicative of induced conversion of inositol pool to pinitol. Agronomic data were taken in every generation from T_2_ to T_5_, confirming viability of the seeds in each generation as assessed by their germination potential (Supplementary Tables S4b–S6b).

Transcriptome analysis of PcINO1-transgenics through microarray presented in Fig. 8 show that 94 pathways up-regulated in all three sets are the pathways that are generally induced in presence of salt. Among them, the 71 unique pathways in PcINO1-transgenics are possibly the ones that were up-regulated due to increased inositol production affecting the salinity tolerance achieved.

Nine genes (Supplementary Table S9) up-regulated in all comparative sets are rather unrelated and less characterized. However, their broad participation in about 94 pathways indicates their widespread regulatory or signaling role in general stress-response. We pulled the previously reported expression data of these 9 genes from Genevisible, an expression search engine powered by Genevestigator. Functionality and localization of these 9 genes have been described in Supplementary Table S7 while the Genevisible expression hits are given. All 9 genes may be related to common signaling in response to stress.

In conclusion, the three types of single copied homozygous salt tolerant transgenic IR-64 rice lines with *PcINO1*, *PcIMT1* and a combination of the two, with identified site of integration exhibiting salt tolerance without perceptible growth compromise, is indicative of the potential for manipulation of the inositol metabolic pathway. Although a number of metabolic pathways are influenced by overexpression of inositol production in the PcINO1-transgenics, not all inositol downstream processes are highly enriched, suggesting that changes in signaling and transcription may also bring the phenotypic superiority. Indeed, after catalytic activity, nucleotide binding and oxidoreductase activities are the most enriched, which suggests possibility of transactivation and signaling roles of inositol overproduction and subsequent reactions that follow leading to salt-tolerance as in the transgenic plants described here.

## Materials and Methods

### Allergenicity Test

Online database *AllergenOnline Database v15 (January 12, 2015*) (http://AllergenOnline.com/) where less than 50% identity with allergenic proteins predicts non-allergenicity^65^, had been used to check the allergenicity of PcIMT1 and PcINO1 proteins.

### SEM Analysis

10, 20 and 30 days old IR-64 calli, obtained from Chinsurah Rice Research Station, West Bengal were transferred separately to a fixative solution (2% glutaraldehyde and 2% paraformaldehyde in 0.1 M cacodylate buffer, pH 7.2) and 3% sucrose, and fixed for 24 h^66^. Fixed tissues were rinsed twice in 0.1 M cacodylate buffer (pH 7.2), dehydrated in graded ethanol series between 30–100% (10 min each), critical point-dried in an AUTOSAMDRI®-815 Series, and were put on metal stubs with gold–palladium ions in a Denton Desk V vacuum deposition system (Denton Vacuum, LLC, Moorestown, NJ, USA). Samples were then examined using a SEM (JSM-6610, JEOL Brasil Instrumentos Científicos Ltda., São Paulo, SP, Brazil) equipped with an energy-dispersive X-ray spectrometer.

### Construction of plant transformation vectors

#### pCAMBIA 1301 vector with *PcINO1/OsINO1* gene

*PcINO1* gene (Accession No: AF412340; 1500 bp; coding *myo*-inositol phosphate synthase) from *Porteresia coarctata*^24^, collected from Sundarban area of West Bengal and *OsINO1* gene from IR-64 rice was restriction digested to generate *Xba*I-*Kpn*I fragment and cloned in pCAMBIA1301 vector (Fig. 1a) at *Xba*I/*Kpn*I site of its MCS under *CAMV35S* promoter (*Hind*III/*Bam*HI) to generate pCAMBIA1301-*Pc/OsINO1* (Fig. 1b).

#### pCAMBIA1301 vector with *PcIMT1* gene

*PcIMT1* gene (Accession No: EU240449; 1100 bp; coding inositol methyl transferase) from *Porteresia coarctata*^17^ was re-amplified with ALM-262 & ALM-263 primers (Supplementary Table S2) to generate *Nco*I(5′)-*Pml*I(3′) flanking ends. Replacement of *gusA* gene at *Nco*I/*Pml*I site in pCAMBIA1301vector with *PcIMT1* gene generated the pCAMBIA1301-*PcIMT1* vector (Fig. 1c) for plant transformation.

#### pCAMBIA1301 vector with *PcIMT1* gene and *PcINO1* gene

In pCAMBIA1301-PcIMT1 vector, *PcINO1/OsINO1*gene (1.5 kb) along with *CAMV35S* promoter (0.8 kb) and *nos* terminator (0.3 kb) altogether were ligated at its MCS. The resulting vector pCAMBIA1301-*PcIMT1* + *PcINO1* (DC1) contained both *PcIMT1* and *PcINO1*gene (Fig. 1d). All the constructs (Fig. 1b–d) were verified through sequence analysis and mobilized in *Agrobacterium* LBA4404 strain by freeze-thaw method^67^.

Empty vector of pCAMBIA1301 has also been used for rice transformation followed by consecutive molecular analysis. The empty-vector transformed plants behaved exactly the same like that of the untransformed IR-64 control lines under stress. Hence, we presented untransformed IR-64 lines as the control for all the following experimentation.

### Standardization of rice transformation protocol

20 days old calli from Elite *indica* rice cultivar IR-64, obtained from Chinsurah Rice Research Station, West Bengal were taken for every transformation experiment. Modified steps of rice transformation protocol^68^ adopted for the study is described in a flow-chart (Supplementary Fig. S4). MS^69^ was used as basic nutrient medium for plant tissue culture. For callus induction 2,4-D (2.5 mg/L) supplementation was applied to the scutellar tissue, incubated in dark for 9 days at 28 ± 1 °C. For regeneration, NAA and BAP were used at 1:5 ratios respectively under 16 hrs/8 hrs photoperiod.

### PCR screening of putative transformants

Genomic DNA from fresh leaves of putative transgenic and untransformed plant lines were isolated by following CTAB method^70^. PCR for presence of *hptII*/*PcIMT1*/*PcINO1* gene in putative transformants were carried out with gene-specific primers ALM-68 & ALM-69, ALM-262 & ALM-263 and ALM-38 & ALM-39 respectively (Supplementary Table S2). PCR reactions were performed in BioRad PCR system using 0.625U of Taq DNA polymerase from Roche Molecular Biochemicals.

### Southern analysis of transformed rice lines

About 10 µg of genomic DNA was isolated from young fresh leaves of putative T_2_ transformants along with untransformed rice leaves, digested with *Hind*III restriction enzyme and subsequently separated on 0.8% agarose gel (USB). DNA fragments from agarose gel were transferred onto Hybond-N^+^ nylon membrane (Amersham Pharmacia Biotech) using vacuum blotter (Bio-Rad). The blot was soaked in 6XSSC, air dried and cross linked using UV-crosslinker (Amersham Biosciences).

~300 bp PCR product from *PcIMT1* gene, amplified by primers, ALM350 and ALM351 was radiolabelled with α- [^32^P]-dCTP to use as probe. Likewise, 600 bp DNA fragment of *PcINO1* part-gene digested with *Bam*HI and 1000 bp DNA fragment containing *hptII* full-length gene from plasmid pCAMBIA1301-*PcINO1*digested with *Xho*I were radiolabelled to use as probe. Prime labeling system of Thermo Scientific has been used for radiolabelling the DNA. Southern experiment had been carried out as par the standard protocol^71^.

### Physiological assay of transformants against salt stress

Germinated plantlets (with proper rooting) of 3 weeks were placed in ½ MS medium containing different salt (NaCl) concentrations (0 mM, 100 mM, 200 mM, 300 mM) for 7 days. Experiment was repeated with best responsive nine independent transgenic lines (three from each type) in replicates to determine their root-behavior, shoot-length, root-length and fresh-weight. After measuring fresh weights, same plants were dried at 60 °C oven for 15 days, dry weights were measured. Replicate lines were withdrawn from salt-stress after 10 days and kept under no-salt condition for 15 days to check the recovery status of the selected plant lines.

### Test for measurement of chlorophyll content

0.5 g of leaf samples were taken and homogenized in presence of liq. N_2_ and 10 ml methanol. Mixtures were centrifuged for 10,000 rpm for 15 min at 4 °C to separate out the supernatant. To make a standard initial mixture from each sample, 0.5 ml of mixtures were taken individually and mixed with 4.5 ml of methanol. O.D were measured at 663 nm and 645 nm to analyze the mixture for Chlorophyll-a and chlorophyll-b respectively. Formula to determine the value of estimated chlorophyll was done by following the method of Nayek *et al*.^72^.

### Transcript analysis

Total RNA were extracted from fresh leaves with RNA-Xpress^TM^ Reagent (Hi Media). C-DNAs were prepared using and following manufacturer’s instruction of kit (Thermo Scientific). RT-PCR was performed with Taq DNA polymerase from NEB. All the primers used in RT-PCR experiments are described in Supplementary Table S2.

### Western blotting for transgenic expression of candidate proteins

About 25 µg of total protein from each plant sample was fractionated in 8% SDS-PAGE and transferred to nitrocellulose membrane (BioRad), blocked with 5%(w/v) skimmed milk (Sigma) for 2 hrs. at 37 °C, primary antibody (1:20000) in 1XTBST added for overnight at 4 °C, the blot washed with large volumes of TBST 4–5 times with fresh changes of wash buffer at 15 min. interval. Then the membrane was immuno-probed with a secondary antibody raised as goat anti-rabbit at 1:5000 (v/v) dilution in TBST [1XTBS and 0.1% (v/v) Tween 20] for 2 hrs. at room temperature, washed with large volumes of TBST 2 times with fresh changes of wash buffer every 10 min and incubated with horseradish peroxidase (HRP)-linked luminescence in dark for few minutes and developed with a florescent ECL detection kit reagent (Amersham Pharmacia Biotech, Little Chalfont, Bucks, UK) following manufacturer’s instruction. Fluorescence was detected by exposing the thoroughly washed membrane to KODAK [X-O(MAT)AR] film (Rochester, NY, USA).

### Identification and estimation of *myo-*inositol and pinitol in transgenic rice

~ 200 mg dried tissues were used for sugar extraction. Ground materials were extracted using methanol:chloroform:water in 12:5:3 (v/v) ratio at 75 °C. Xylitol was used as internal standard. Extracts were de-ionised through resin bed (Dowex 50 W H +/Dowex 1\8 formate; Dow Chemical Co., Pevely, MA, USA), lyophilized and stored at −20 °C. Trimethylsilyl derivatives of inositols and inositol O-methyl-ethers were separated by capillary gas chromatography as previously described^73^.

### Detection of site of introgression in transgenic plant lines

Hi-TAIL PCR^27^ method utilizes nested known sequence specific primers with melting temperature (Tm) > 65 °C in consecutive reactions together with a short (15–16 nucleotides) arbitrary degenerate (AD) primer (T_m_ 45 °C) with 64–256 folds of degeneracy, so that relative amplification efficiencies of target and non-target products can be thermally controlled.

### Photosynthetic efficiency analysis of transgenic lines

Measurements were taken from middle part of apical and second apical leaf using PHOTOSYNTHETIC EFFICIENCY ANALYZER (Hansatech Instruments Ltd., King’s Lynn, UK) and recorded upto 1 sec,with data acquisition every 10 μS, for 1 sec using a single flash of light intensity 3000 μmol/m^2^/sec without pre-illumination. The values are taken at 0.05 ms (T1), 0.10 ms (T2), 0.30 ms (T3), 2 ms (T4) and 30 ms (T5) for 1 sec.^74^. Each chlorophyll-a fluorescence transient O-J-I-P was analyzed according to the JIP-test^74^. The data was analyzed using HANSATECH BIOLYZER SOFTWARE.

### Agronomic character analysis

Selected plants were grown to maturity in greenhouse and self-pollinated. Agronomic data were collected manually. Grain characters (grain wt., grain length, grain width ration) were analyzed by using SMART-GRAIN VERSION 1.1 software.

### Microarray

Total RNA was extracted from leaves of one-month old WT (wild type), OsINO1 and PcINO1– transformed plants either in control condition or under 5 days treatment of 150 mM NaCl for comparative transcriptome study, using Agilent cDNA microarray. Obtained transcripts were subjected to comparative microarray analysis.

## Supplementary information

Supplementary documents

## References

[1] Allen, J. C. & Hamilton, R. J. Rancidity in Foods. 3^rd^ edition. *Aspen Publishers, Inc*. 192–216 (1994).

[2] Serrano, R. *et al*. A glimpse of the mechanisms of ion homeostasis during salt stress. *J. Exp. Bot.* **50**, 1023–1036 (1999).

[3] Verma, A. K. & Singh., D. Abiotic stress and crop improvement: current Scenario. *Advances in Plants & Agriculture Research.* **4**(4), 345–346 (2016).

[4] Zhu, J. K. Plant salt tolerance. *Trends Plant Sc.* **6**, 66–71 (2001). 11173290 10.1016/s1360-1385(00)01838-0

[5] Hasegawa, P. M., Bressan, A. P., Jian-Kang Zhu, J. K. & Bohnert, H. J. Plant cellular and molecular responses to high salinity. *Ann. Rev. Plant Physiol., Plant Mol. Biol.* **51**, 463–499 (2000). 15012199 10.1146/annurev.arplant.51.1.463

[6] Khush, G. S. Green revolution: the way forward. *Nat. Rev. Genet2* **10**, 815–22 (2001). 10.1038/35093585 11584298

[7] Datta, S. K. Rice biotechnology: A need for developing countries. *Ag Bio Forum.* **7**, 31–35 (2004).

[8] Ismail, A. M., Heuer, S., Thomson, M. J. & Wissuwa, M. Genetic and genomic approaches to develop rice germplasm for problem soils. *Plant Mol. Biol.* **65**, 547–570 (2007). 17703278 10.1007/s11103-007-9215-2

[9] Schneider, S. Inositol transport proteins. *FEBS Letters.* **589**(10), 1049–1058 (2015). 25819438 10.1016/j.febslet.2015.03.012

[10] Loewus, F. A. Inositol biosynthesis. *Morré DJ, editor. Inositol metabolism in plants*. 13–19 (1990).

[11] Loewus, F. A. & Murthy, P. P. N. *Myo*-Inositol metabolism in plants. *Plant Science.* **105**(1), 1–19 (2000).

[12] Henry, S. A., Gaspar, M. L. & Jesch, S. A. The response to inositol: regulation of glycerolipid metabolism and stress response signaling in yeast. *Chem. Phys. Lipids* **180**, 23–43 (2014). 24418527 10.1016/j.chemphyslip.2013.12.013 PMC3980031

[13] Stevenson, J. M., Perera, I. Y., Heilmann, I., Persson, S. & Boss, W. F. Inositol signaling and plant growth. *Trends Plant Sc.* **5**, 252–258 (2000). 10838616 10.1016/s1360-1385(00)01652-6

[14] Xue, H., Chen, X. & Li, G. Involvement of phospholipids signaling in plant growth and hormone effects. *Curr. Opin. Plant Biol.* **10**, 483–489 (2007). 17709277 10.1016/j.pbi.2007.07.003

[15] Okada, M. & Yei, K. Nuclear phosphoinositide signaling regulates messenger RNA export. *RNA Biol.* **6**, 12–16 (2009). 19106628 10.4161/rna.6.1.7439 PMC3704435

[16] Sheveleva, E., Chmara, W., Bohnert, H. J. & Jensen, R. G. Increased salt and drought tolerance by d-ononitol production in transgenic *Nicotiana tabacum* L. *Plant Physiol.* **25**, 1211–1219 (1997). 10.1104/pp.115.3.1211 PMC158586 12223867

[17] Sengupta, S., Patra, B., Ray, S. & Majumder, A. L. Inositol methyl tranferase from a halophytic wild rice, *Porteresia coarctata* Roxb. (Tateoka): regulation of pinitol synthesis under abiotic stress. *Plant, Cell Environ.* **31**, 1442–1459 (2008). 18643954 10.1111/j.1365-3040.2008.01850.x

[18] Bennett, M., Onnebo, S. M., Azevedo, C. & Saiardi, A. Inositol pyrophosphates: metabolism and signaling. *Cell Mol Life Sci.* **63**(5), 552–64 (2006). 16429326 10.1007/s00018-005-5446-z PMC11136278

[19] Williams, S. P., Glenda E. Gillaspy, G. E. & Perera, I. Y. Biosynthesis and possible functions of inositol pyrophosphates in plants. *Front Plant Sci*. 10.3389/fpls.2015.00067 (2015). 10.3389/fpls.2015.00067 PMC4325660 25729385

[20] Lorence, A., Chevone, B. I., Mendes, P. & Nessler, C. L. Myo-inositol oxygenase offers a possible entry point into plant ascorbate biosynthesis. *Plant Physiol.* **134**, 1200–1205 (2004). 14976233 10.1104/pp.103.033936 PMC389944

[21] Loewus, F. A. Inositol and plant cell wall polysaccharide biogenesis. *Subcellular Biochem.* **39**, 21–45 (2006). 17121270 10.1007/0-387-27600-9_2

[22] Patra, B., Ray, S., Richter, A. & Majumder, A. L. Enhanced salt tolerance of transgenic tobacco plants by co-expression of *PcINO1* and *McIMT1* is accompanied by increased level of *myo*-inositol and methylated inositol. *Protoplasma.* **245**, 143–52 (2010). 20524018 10.1007/s00709-010-0163-3

[23] Das, P., Nutan, K. K., Pareek, S. S. & Pareek, A. Understanding salinity responses and adopting ‘omics based’ approaches to generate salinity tolerant cultivars of rice. *Front. Plant Sci.* **6**, 712 (2015). 26442026 10.3389/fpls.2015.00712 PMC4563168

[24] Majee, M. *et al*. A novel salt-tolerant L-myo-Inositol-1-phosphate synthase from *Porteresia coarctata* (Roxb.) Tateoka, a halophytic wild rice. *J. Biol. Chem.* **279**, 28539–28552 (2004). 15016817 10.1074/jbc.M310138200

[25] Hiei, Y., Komari, T. & Kubo, T. Transformation of rice mediated by *Agrobacterium tumefaciens*. *Plant Mol. Biol.* **35**, 205–218 (1997). 9291974

[26] Nishimura, A., Aichi, I. & Matsuoka, M. A protocol for Agrobacterium-mediated transformation in rice. *Nat. Protl.* **1**, 2796–2802 (2007). 10.1038/nprot.2006.469 17406537

[27] Liu, Y. G. & Chen, Y. High-efficiency thermal asymmetric interlaced PCR for amplification of unknown flanking sequences. *BioTechniques.* **43**, 649–656 (2007). 18072594 10.2144/000112601

[28] Krasensky, J. & Jonak, C. Drought, salt, and temperature stress-induced metabolic rearrangements and regulatory networks. *J. Exp. Bot.* **63**, 1593–1608 (2012). 22291134 10.1093/jxb/err460 PMC4359903

[29] Gupta, B. & Huang, B. Mechanism of salinity tolerance in plants: physiological, biochemical, and molecular characterization. *International Journal of Genomics*. 10.1155/2014/701596 (2014). 10.1155/2014/701596 PMC3996477 24804192

[30] Vernon, D. M., Ostrem, J. A. & Bohnert, H. J. Stress perception and response in a facultative halophyte: the regulation of salinity-induced genes in *Mesembryanthemum crystallinum*. *Plant Cell Environ.* **16**, 437–444 (1993).

[31] Sengupta, S. *et al*. Manipulation of inositol metabolism for improved plant survival under stress: a network engineering approach. *J. Plant Biochem. Biotechnol.* **21**, 15–23 (2012).

[32] Salvi, P. *et al*. Differentially expressed galactinol synthase(s) in chickpea are implicated in seed vigor and longevity by limiting the age induced ROS accumulation. *Sci. Rep*, 10.1038/srep35088 (2016). 10.1038/srep35088 PMC5057127 27725707

[33] Duana, J. *et al*. OsMIOX, a myo-inositol oxygenase gene, improves drought tolerance through scavenging of reactive oxygen species in rice (*Oryza sativa* L.). *Plant Sci.* **196**, 143–151 (2012). 23017909 10.1016/j.plantsci.2012.08.003

[34] Alok, A. *et al*. Biochemical characterization and spatio-temporal expression of myo-inositol oxygenase (MIOX) from wheat (*Triticum aestivum* L.). *Plant Gene.* **4**, 10–19 (2015).

[35] Krishnamoorthy, P., Sanchez-Rodriguez, C., Heilmann, I. & Persson, S. Regulatory roles of phosphoinositides in membrane trafficking and their potential impact on cell-wall synthesis and re-modelling. *Ann. Bot.* **114**, 1049–1057 (2014). 24769536 10.1093/aob/mcu055 PMC4195552

[36] Majumder, A. L., Johnson, M. D. & Henry, S. A. 1L-myo-inositol-1-phosphate synthase. *Biochim. Biophys. Acta.* **1348**, 245–256 (1997). 9370339 10.1016/s0005-2760(97)00122-7

[37] Majumder, A. L., Chatterjee, A., Ghosh Dastider, K. & Majee, M. Diversification and evolution of L-myo-inositol 1-phosphate synthase. *FEBS Lett.* **533**, 3–10 (2003). 10.1016/s0014-5793(03)00974-8 14550537

[38] Basak, P. *et al*. An evolutionary analysis identifies a conserved pentapeptide stretch containing the two essential lysine residues for rice L-myo-inositol 1-phosphate synthase catalytic activity. *PLoS One*, 10.1371/journal.pone.0185351 (2017). 10.1371/journal.pone.0185351 PMC5614600 28950028

[39] Mishra, A. & Tanna, B. Halophytes: Potential Resources for Salt Stress Tolerance Genes and Promoters. *Front. Plant Sci*. 10.3389/fpls.2017.00829 (2017). 10.3389/fpls.2017.00829 PMC5435751 28572812

[40] RayChoudhuri, A. & Majumder, A. L. Salinity induced enhancement of L-*myo*-inositol 1-phosphate synthase in rice (*Oryza sativa* L.). *Plant Cell Environ.* **19**, 1437–1442 (1996).

[41] GhoshDastidar, K., Chatterjee, A., Chatterjee, A. & Majumder, A.L. Evolutionary Divergence of L-myo-Inositol 1-Phosphate Synthase: Significance of a Core Catalytic Structure. Book chapter: *Biology of Inositols and Phosphoinositides*. 313–338 (2006). 17121281

[42] Das-Chatterjee, A. *et al*. Introgression of a novel salt-tolerant L-*myo*-inositol 1-phosphate synthase from *Porteresia coarctata* (Roxb.) Tateoka (*PcINO1*) confers salt tolerance to evolutionary diverse organisms. *FEBS Lett.* **580**, 3980–3988 (2006). 16806195 10.1016/j.febslet.2006.06.033

[43] Vernon, D. M. & Bohnert, H. J. A Novel Methyl Transferase Induced by Osmotic Stress in the Facultative Halophyte M. Crystallinum. *EMBO J.* **11**, 2077–2085 (1992a). 1600940 10.1002/j.1460-2075.1992.tb05266.x PMC556674

[44] Vernon, D. M. & Bohnert, H. J. Increased expression of a *myo*-inositol methyl transferase in *Mesembryanthemum crystallinum* is part of a stress response distinct from Crassulacean acid metabolism induction. *Plant Physiol.* **99**, 1695–1698 (1992b). 16669095 10.1104/pp.99.4.1695 PMC1080685

[45] Visarada, K. B. R. S., Sailaja, M. & Sarma, N. P. Effect of callus induction media on morphology of embryogenic callus in rice genotypes. *Biol. Plant.* **45**, 495–502 (2002).

[46] Nabors, M. W., Heyser, J. W., Dykes, T. A. & Demott, K. J. Long-duration, high frequency plant regeneration from cereal tissue cultures. *Planta.* **157**, 385–391 (1983). 24264334 10.1007/BF00397195

[47] Bevitori, R., Popielarska-Konieczna, M., dos Santos, E. M., Grossi-de-Sá, M. F. & Petrofeza, R. Morpho-anatomical characterization of mature embryo-derived callus of rice (Oryza sativa L.) suitable for transformation. *Protoplasma.* **251**, 545–554 (2014). 24085343 10.1007/s00709-013-0553-4

[48] Wang, Y., Li, K. & Li, X. Auxin redistribution modulates plastic development of root system architecture under salt stress in *Arabidopsis thaliana.**J Plant Physiol.* **166**, 1637–1645 (2009). 19457582 10.1016/j.jplph.2009.04.009

[49] Boursier, P. & Läuchli, A. Growth responses and mineral nutrient relations of salt-stressed sorghum. *Crop Sci.* **30**, 1226–1233 (1990).

[50] Asch, F., Dingkuhn, M., Sow, A. & Audebert, A. Drought-induced changes in rooting patterns and assimilate partitioning between root and shoot in upland rice. *Field Crop Res.* **93**, 223–236 (2005).

[51] Zolla, G., Heimer, Y. M. & Barak, S. Mild salinity stimulates a stress-induced morphogenic response in *Arabidopsis thaliana* roots. *J. Expt. Bot.* **61**, 211–224 (2010). 10.1093/jxb/erp290 PMC2791118 19783843

[52] Horie, T., Karahara, I. & Katsuhara, M. Salinity tolerance mechanisms in glycophytes: An overview with central focus on rice plants. *Rice.* **5**, 1–11 (2012). 27234237 10.1186/1939-8433-5-11 PMC5520831

[53] Sun, F. *et al*. Salt modulates gravity signalling pathway to regulate growth direction of primary roots in *Arabidopsis*. *Plant Physiol.* **146**, 178–188 (2008). 18024552 10.1104/pp.107.109413 PMC2230569

[54] Wang, Y. *et al*. Salt-induced plasticity of root hair development is caused by ion disequilibrium in *Arabidopsis thaliana*. *J. Plant Res.* **121**, 87–96 (2008). 18060349 10.1007/s10265-007-0123-y

[55] Ji, H. *et al*. The salt overly sensitive (SOS) pathway: established and emerging roles. *Molecular Plant* **6**, 275–286 (2013). 23355543 10.1093/mp/sst017

[56] Galvan-Ampudia, C. S. & Testerink, C. Salt stress signals shape the plant root. *Curr. Opin. Plant Biol.* **14**, 296–302 (2011). 21511515 10.1016/j.pbi.2011.03.019

[57] Sarkar, T., Thankappan, R., Kumar, A., Mishra, G. P. & Dobaria, J. R. Heterologous Expression of the AtDREB1A Gene in Transgenic Peanut-Conferred Tolerance to Drought and Salinity Stresses. *PLoS One* **9**, e110507 (2014). 25545786 10.1371/journal.pone.0110507 PMC4278701

[58] Rai, A. C., Singh, M. & Shah, K. Engineering drought tolerant tomato plants over-expressing BcZAT12 gene encoding a C 2H2zinc finger transcription factor. *Phytochem.* **85**, 44–50 (2013). 10.1016/j.phytochem.2012.09.007 23079765

[59] Farooq, M., Wahid, A., Kobayashi, N., Fujita, D. & Basra, S. M. A. Plant drought stress: effects, mechanisms and management. *Agron Sustain Dev.* **29**, 185–212 (2009).

[60] Jami, S. K. *et al*. Ectopic expression of an annexin from Brassica juncea confers tolerance to abiotic and biotic stress treatments in transgenic tobacco. *Plant Physiol Biochem.* **46**(12), 1019–30 (2008). 18768323 10.1016/j.plaphy.2008.07.006

[61] Ravikumar, G. *et al*. Stress-inducible expression of AtDREB1A transcription factor greatly improves drought stress tolerance intransgenic Indica rice. *Transgen. Res.* **23**, 421–439 (2014). 10.1007/s11248-013-9776-6 PMC4010723 24398893

[62] Kalaji, M. H. & Pietkiewicz, S. Salinity Effects on Plant Growth and Other Physiological Processes. *Acta Physiol. Plant.* **143**, 89–124 (1993).

[63] Kalaji, H. M. & Nalborczyk, E. Gas Exchange of Barley Seedlings Growing under Salinity Stress. *Photosynthetica.* **25**, 197–202 (1991).

[64] Kalaji, H. M., Govindjee., Bosa, K., Koscielniak, J. & Zuk-Golaszewska, K. Effects of salt stress on photosystem II efficiency and CO_2_ assimilation of two Syrian barley landraces. *Environ. Exp. Bot.* **73**, 64–72 (2011).

[65] Aalberse, R. C. Structural biology of allergens. *J Allergy Clin Immunol.* **106**, 228–38 (2000). 10932064 10.1067/mai.2000.108434

[66] Karnovsky, M. J. A formaldehyde-glutaraldehyde fixative of high osmolality for use in electron-microscopy. *J. Cell Biol.* **27**, 137A (1965).

[67] An, G., Ebert, P. R., Mitra, A. & Ha, S. B. Binary vectors. *Plant Molecular Biology Manual*. 1–19 (1988).

[68] Hiei, Y., Ohta, S., Komari, O. & Takashi, K. Efficient transformation of rice (*Oryza sativa* L.) mediated by *Agrobacterium* and equence analysis of the boundaries of the T-DNA. *The Plant Journal.* **6**(2), 271–282 (1994). 7920717 10.1046/j.1365-313x.1994.6020271.x

[69] Murashige, T. & Skoog, F. A revised medium for rapid growth and bioassays with tobacco tissue cultures. *Physiol. Plantarum.* **15**, 473–497 (1962).

[70] Doyle, J. J. & Doyle, J. L. Isolation of plant DNA from fresh tissue. *Focus* **12**, 13–15 (1990).

[71] Southern, E. Detection of Specific Sequences Among DNA Fragments Separated by Gel Electrophoresis. *J. Mol.Biol.* **98**, 503–517 (1975). 1195397 10.1016/s0022-2836(75)80083-0

[72] Nayek, S., Choudhury, I. H., Jaishee, N. & Roy, S. Spectrophotometric analysis of chlorophylls and carotenoids from commonly grown fern species by using various extracting solvents. *Res. J. Chem. Sc.* **4**, 63–69 (2014).

[73] Peterbauer, T. & Richter, A. Galactosylononitol and stachyose synthesis in seeds of adzuki bean. Purification and characterization of stachyose synthase. *Plant Physiol.* **117**, 165–72 (1998). 9576785 10.1104/pp.117.1.165 PMC34999

[74] Strasser, B. J. & Strasser, R. J. Measuring fast fluorescence transients to address environ-mental questions: The JIP test. *Mathis, P. (ed.) Photosynthesis: From Light to Biosphere* **5**, 977–980 (1995).

