# Author Correction: Selective manipulation of the inositol metabolic pathway for induction of salt-tolerance in *indica* rice variety

**DOI:** 10.1038/s41598-025-19785-y

**Published:** 2025-09-17

**Authors:** Rajeswari Mukherjee, Abhishek Mukherjee, Subhendu Bandyopadhyay, Sritama Mukherjee, Sonali Sengupta, Sudipta Ray, Arun Lahiri Majumder

**Affiliations:** 1 Division of Plant Biology, Bose Institute, Kolkata, India; 2 Department of Botany, Bethune College, Kolkata, India; 3 School of Plant Environment and Soil Sciences, Lousiana State University Agricultural Center, Lousiana, USA; 4 Department of Botany, Centre of Advanced Studies, University of Calcutta, Kolkata, India

Correction to: *Scientific Reports* 10.1038/s41598-019-41809-7, published online 29 March 2019

This Article contains errors in the Supplementary Information file.

As a result of errors during figure assembly, in Figure S7d an image for 4PcINO1/300 was a duplication of an image for 4PcINO1/400. The corrected Figure S7 and accompanying legend is included below.

**Figure S7:**
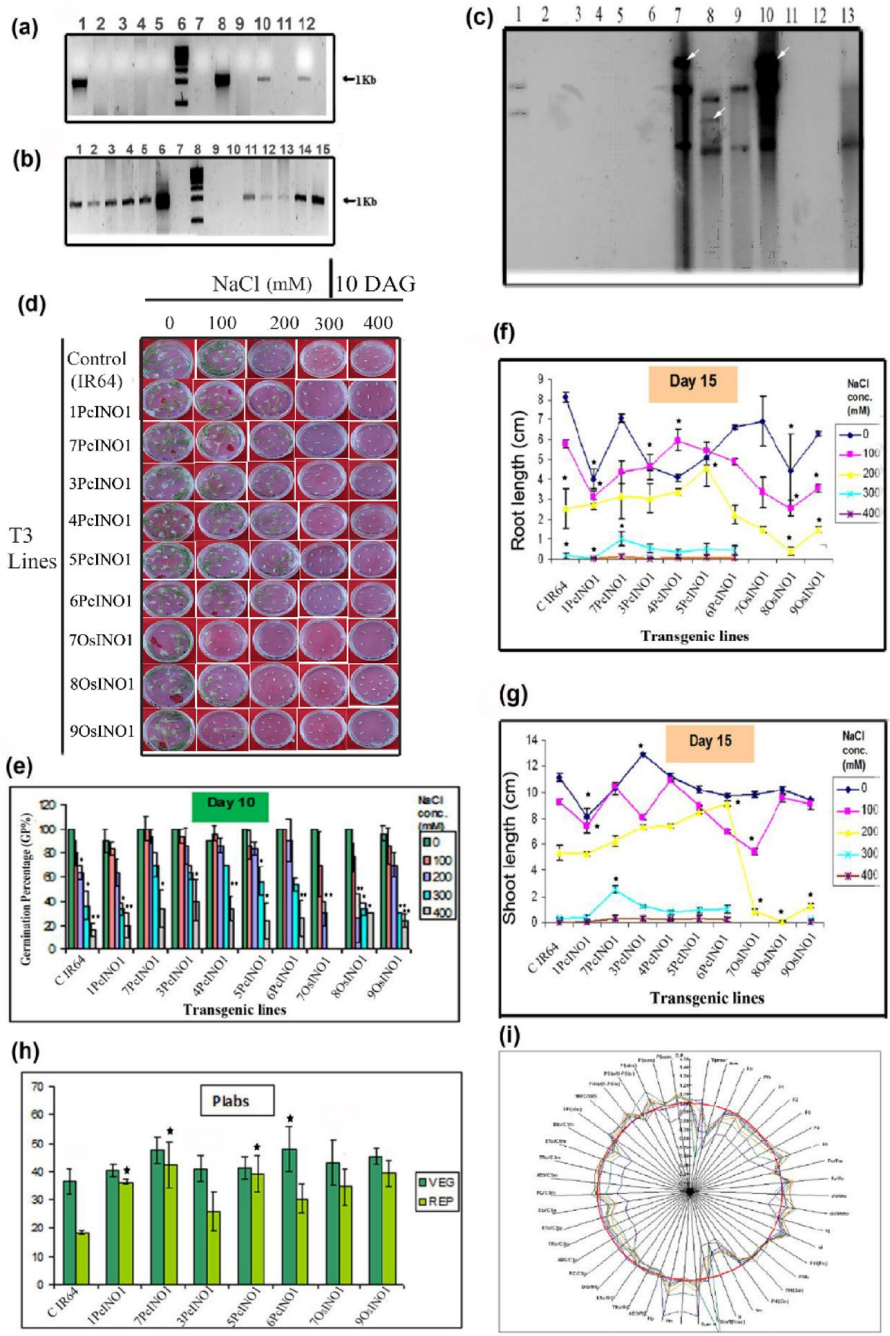
Representative pictures showing generation and selection of *OsINO1* transformants followed by comparative salt-tolerant experiments with *PcINO1* lines based on phenotypic analysis under salt stress. (a) and (b) Representing PCR profiles of *OsINO1* overexpressing transformants exhibiting the presence of *hptII* gene. (c) Representative autoradiogram exhibiting Southern blot hybridization of different T_3_ PCR positive *OsINO1* transformants probed with α-P^32^ labelled ~ 600bp *BamHI* digested DNA fragment from the coding region common to both *PcINOI/OsINO1* gene cloned in pCAMBIA-1301 vector. White arrows are indicative of transgene insertions. (d) and (e) Comparative study on effect of salt (NaCl) on Germination Percentage (GP %) of *PcINO1/OsINO1* transgenics on 10 DAG (Days After Germination) with control (rice IR64) grown on MS medium. (f) and (g) Comparative evaluation on effect of salt (NaCl; 0, 100, 200, 300 and 400 mM) on shoot and root length (cm) of *PcINO1/OsINO1* transgenics (T3) and control (rice IR64) grown on MS medium for 15 days. (h) PIabs value of the transgenic lines (*PcINO1/OsINO1*) and control plant (IR64) in their vegetative and reproductive stages respectively. (i) Radar-plot presentation of photosynthetic parameters quantifying the behaviour of PSII in Rice transgenic plants (*PcINO1/OsINO1*) compared with the control (rice var.IR64). Biological samples are taken in triplicates. Data represented average of three replica sets ± SD (P ≤ 0.05).

