# Supplementary material for: Selective manipulation of the inositol metabolic pathway for induction of salt-tolerance in indica rice variety
Source: Sci Rep. 2019 Mar 29;9:5358. doi: 10.1038/s41598-019-41809-7 (PMC6441109; doi:10.1038/s41598-019-41809-7)
Supplement: Supplementary file 1 — Supplementary documents [file 41598_2019_41809_MOESM1_ESM.pdf]

TITLE

Selective manipulation of the inositol metabolic pathway for induction of salt-tolerance in *indica* rice variety.

AUTHORS

Rajeswari Mukherjee[1], Abhishek Mukherjee[1], Subhendu Bandyopadhyay[1,*], Sritama Mukherjee[1,2,*], Sonali Sengupta[1,3], Sudipta Ray[1,4] and Arun Lahiri Majumder[1, †]

```
              130       140       150       160       170       180       190       200
PcIMT  QGSLGPLLVLHHDKVMMESWFHLNDYILEGGVPFKRAHGMIQFDYTGTDERFNHVFNQGMAHHTILVMKKLLDNYNGFND
                                               .::   ..    .. ..: ::..:. .:.:       .:.
gi|510 FTSSAQVSTFKDLANAFVQKGHTAFIVDWSEAACTDGLPGVQFAEYNAAASNTYDIGQLMAKYTVDLMNK---CKIPLNN
            90       100       110       120       130       140       150          160

              210       220       230       240         250       260       270
PcIMT  VKVLVDVGGNIGVNVSMIVAKHTHIKGINYDLPHVVADAPSYP--GVEHVGGNMFESIPQADAIFMKWVLHDWSDEHCVK
         ..    :: ..: .:  ..:::.. : ::  .:...:  :. :  : .. :  . .:  .  ..:   .:
gi|510 IQY---VGHSLGSHVCGFAAKHVK-KLINKTMPYILALDPADPSFGSNKCGERICKSDAKRIVVFKTSILGIGENIIGHL
                 170       180        190       200       210       220       230

     280       290       300       310       320       330       340       350
PcIMT  ILNKCYESLAKGGKIILVESLIPVIPEDNLESHMVFSLDCHTLVHNQGGKERSKEDFEALASKTGFSTVDVICCAYDTWV

gi|510 LIVFDGGKSQPACSWYDVPCSHSESIVYATGMVSGRCQHLAVPWTAQQRINPIQWKFWRVFTSNIPAYPTSDTTNCVVLN
        240       250       260       270       280       290       300       310
```

Supplementary Figure S1

```
            90       100       110       120         130       140       150       160
PcIMT  RLLASHSVLTCKLQKGEGGSQRVYGPAPLCNYLASNDGQGSLG--PLLVLHHDKVMMESWFHLNDYILEGGVPFKRAHGM
                                               :.:  :. .   : . :..    : .: ....:   . :.
gi|810 VHLGNGGPCLFMRKVSHVILHGLHIHSCNTSVLGDVLVSESIGVEPVHAQDGDAITMRNV--TNAWIDHNSLP-DCSDGL
     120       130       140       150       160       170         180       190

               170       180       190       200          210       220       230
PcIMT  IQ--FDYTGTDERFNHVFNQGMAHHTILVMKKLLDNYNGFNDVKVLV---DVGGNIGVNVSMIVAKHTHIKGINYDLPHV
       :.  .. ::     :: ::    :: .... .  :.:.  ...:: :   . : : :  .       .:. . :::  ..
gi|810 IDVTLSSTGITISNNHFFN----HHKVMLLGHD-DTYDDDKSMKVTVAFNQFGPNAGQRMPRARYGLVHVANNNYDQWNI
        200       210           220        230       240       250       260       270

         240       250       260       270       280       290       300       310
PcIMT  VA-DAPSYPGVEHVGGNMFESIPQADAIFMKWVLHDWSDEHCVKILNKCYESLAKGGKIILVESLIPVIPEDNLESHMVF
        :  . : : .   :..
gi|810 YAIGGSSNPTILSEGNSFTAPNENYKKEVTKRIGCESTSACANWVWRSTRDAFSNGAYFVSSGKTEETNIYNSNEAFKVE
     280   290  300   310  320   330  340  350
```

Supplementary Figure S2

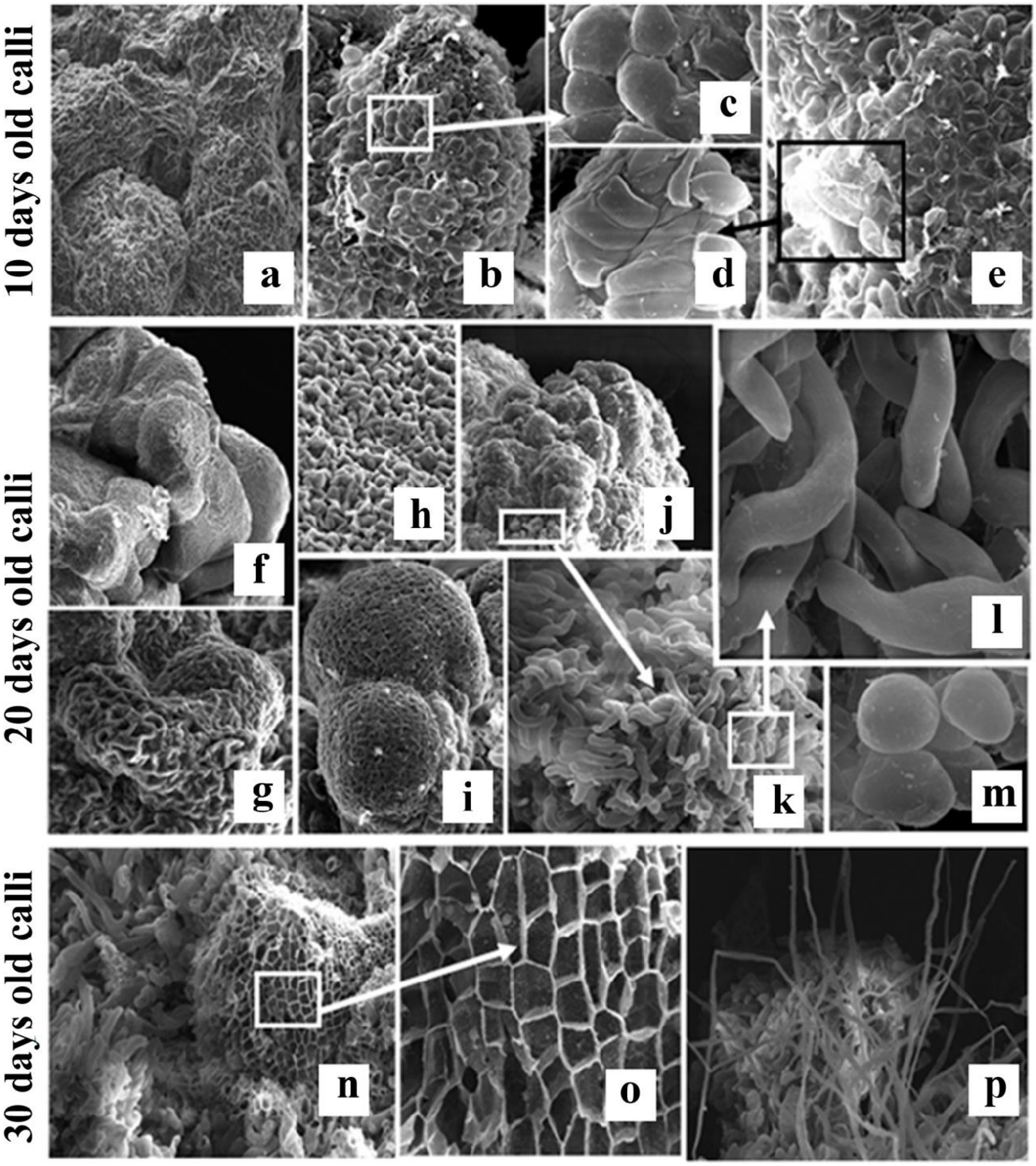

Supplementary Figure S3

Explant: 15-days old actively growing embryogenic calli

↓

Explants in *EM media is supplemented with 2mg l^{-1} 2,4-D

↓ **24 hrs precondition**

Infection of explants with overnight grown culture of *Agrobacterium* (strain LBA4404) supplemented with 150 µM Acetosyringone

30 mins ↓ **infection at 0.8 O.D.**

Explants on co-cultivation medium supplemented with 150 µM Acetosyringone

2 days ↓ **Co-cultivation**

Transferred to EM medium with cefotaxim (300 mg l^{-1})

↓ **7 days**

Transferred to EM medium with cefotaxim (250mg l^{-1}) and hygromycin (50mg l^{-1})

4 weeks ↓ **Callus selection**

Recovery of the selected calli in EM medium

1-2 weeks ↓ **Embryo formation**

Embryogenic calli transferred to *RM medium

3-4 weeks ↓ **Embryo maturation and regeneration**

Growth of the plantlets

↓

Recovery of the transformant lines from hygromycin selection in liquid 1/2 MS medium

↓

Elongation and rooting of the transformants in liquid 1/2 MS medium

↓

Transferred to soilrite

***EM= Embryogenic media**
***RM= Regeneration media**

Supplementary Figure S4

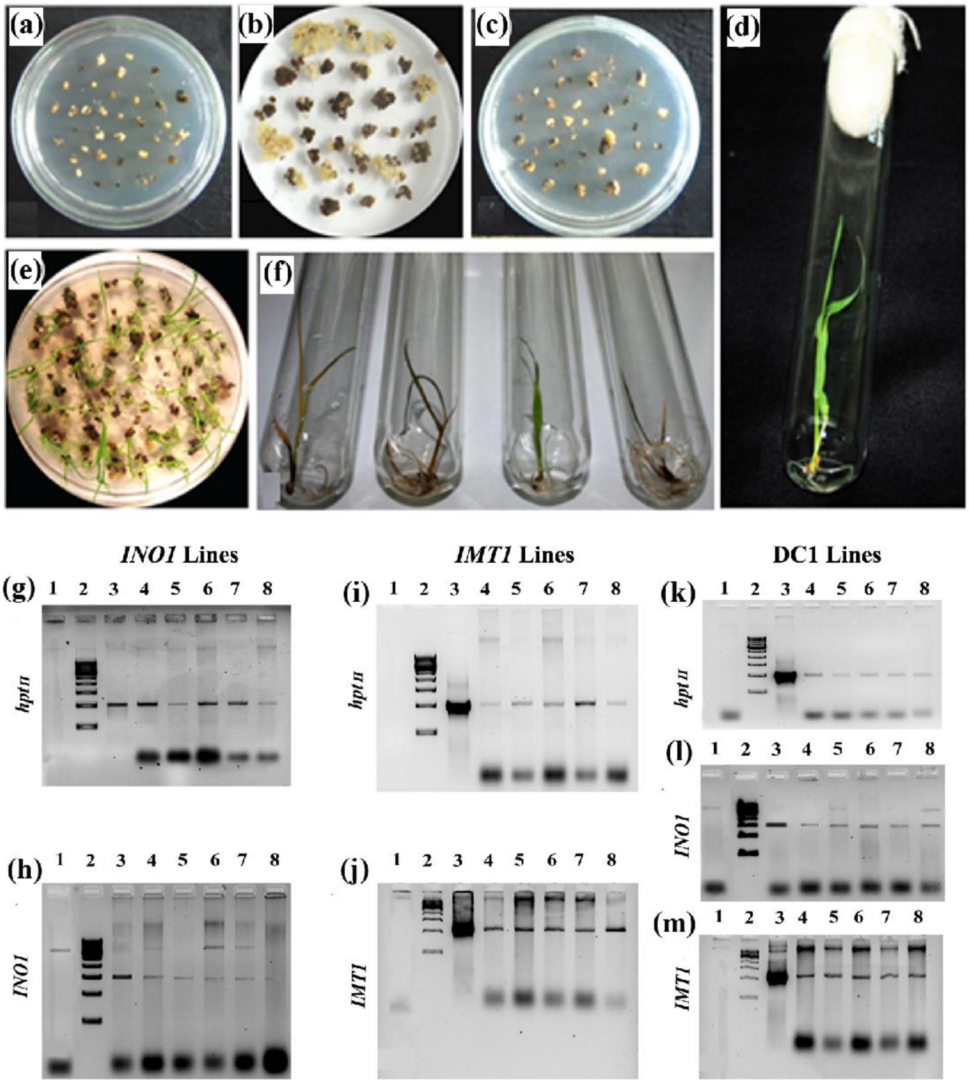

Supplementary Figure S5

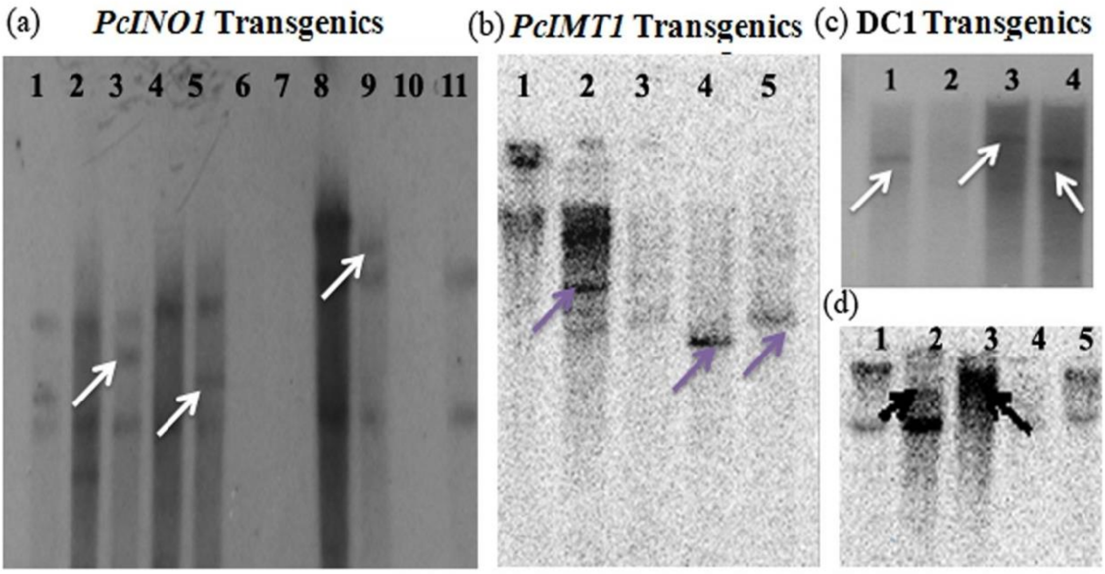

Supplementary Figure S6

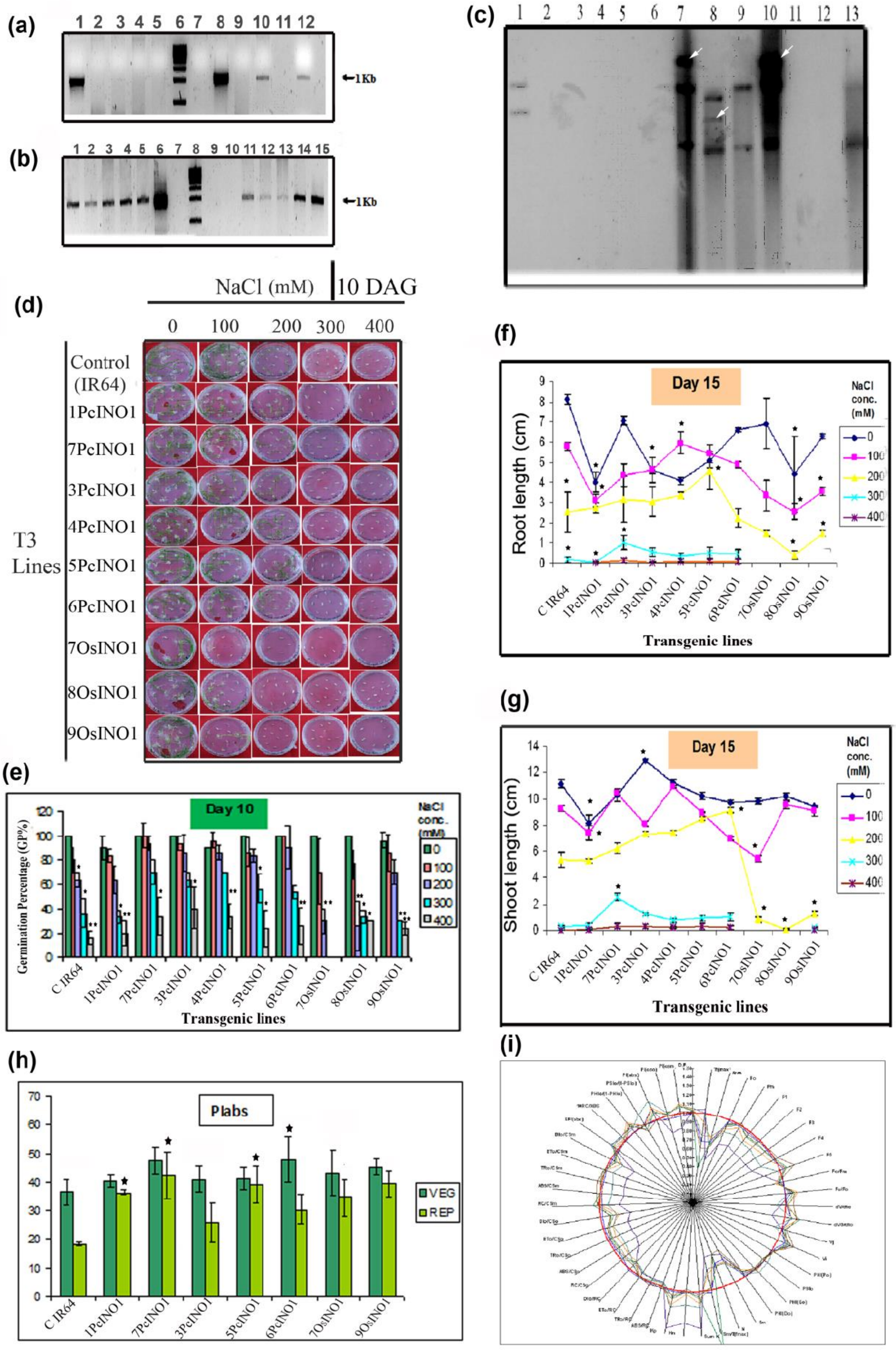

Supplementary Figure S7

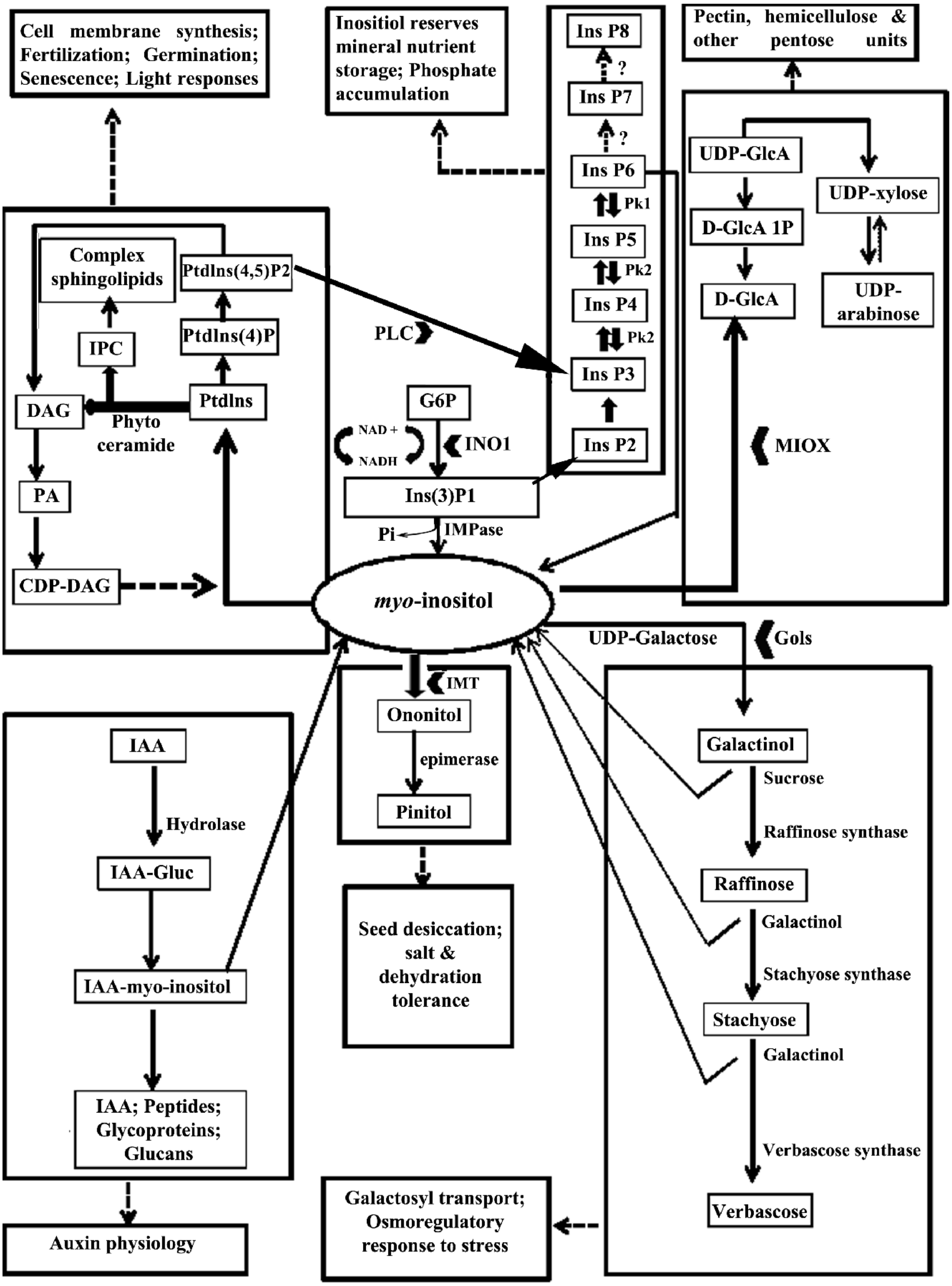

Supplementary Figure S8

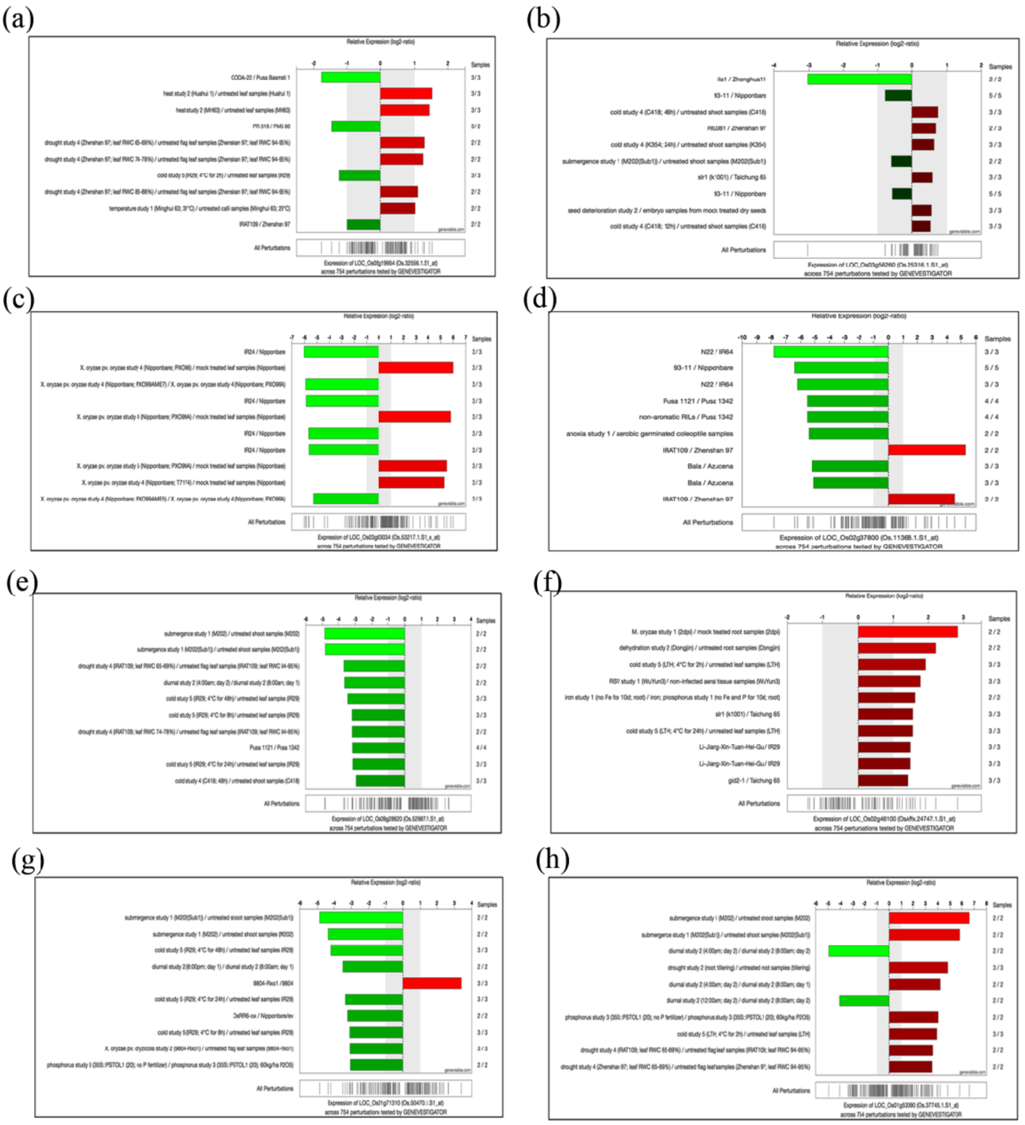

Supplementary Figure S9

Supplementary Table. S1: Site of integration for transgenic lines:

**Types of Transgenics**	**Transgenic Plant Lines**	**Site of Integration**	**Locus match**
PcINO1 integrated IR64 Lines	**Line 7PC**	Chromosome 8<br>(10,377,546bp to 10,377,563bp)	CP012616
PcIMT1 integrated IR64 Lines	**Line 10IMT**	Chromosome 5<br>(10812769bp to 10812782bp)	CP012613
(PcINO1+PcIMT1) integrated IR64 Lines	**Line 6DC1**	Chromosome 5<br>(21357777bp to 21358480bp)	CPO12613.1

Supplementary Table. S2 : Primers used for PCR reactions

Primer name	Primer Sequences	Annealing Temperature	Gene Specificity	Product Size
ALM-68<br>ALM-69	5’ATGAAAAAGCCTGAACTCACCGCG3’<br>5’CTATTTCTTTGCCCTCGGACGAGT3’	55^0C	Hygromycin resistant gene (*hptII*)	1.0 kb
ALM-262<br>ALM-263	5’TTGACCATGGATGACCACCTACACCA3’<br>5’CCACCACGTGCTTCTTGTAGAGCT 3’	58^0C	*PcIMT1* gene	1.1 kb
ALM-38<br>ALM-39	5' ACATATGTTCATCGAGAGCTTCCG3'<br>5’GCTCGAGCTTGTTACTCCAGGATCATGTT 3’	55^0C	*PcINO1* gene	1.5 kb
ALM350<br>ALM350	5’GGATCCATGACCACCTACACCAATGGCAA 3’<br>5’GAG CTT GCATGTTAACACAGAGTAGCTC 3’	56^0C	*PcIMT1*<br>Part gene	288 bp
ALM-106<br>ALM-107	5'ATCCATATGTGGTGCCTCTCCCTGGCATCTATG3’<br>5’ATCAAGCTTTTATCATGATGATCTGCCCCATCTGCTCCT 3’	62^0C	*PcINO1*<br>37 amino acid stretch specific part gene	173 bp
ALM-181<br>ALM-182	5’CCTCATGAAGATCCTGACGG 3’<br>5’GGAATGTGCTGAGAGATGCC 3’	50^0C	*Actin* gene	~500 bp

<u>**Supplementary Table. S3: Phenotypic characters of root**</u>

Plant sample	No. AD. Rt	No. ltrl Rt	Avg. RT density
IR64-0	1.3	3	1.37
IR64-100	9	2.66	0.79
IR64-200	6	1	0.72
IR64-300	8	5.66	0.54
7Pc INO 1-0	2	10.33	2.16
7Pc INO 1-100	4	15.33	1.22
7Pc INO 1-200	9	10	2.02
7Pc INO 1-300	7	10	2.20
10 Pc IMT-0	2.66	17	0.60
10 Pc IMT-100	5.66	17	1.25
10 Pc IMT-200	2	12	1.43
10 Pc IMT-300	5	16	1.66
6 DC 1-0	1.66	8	1.37
6 DC 100	5	8	1.75
6 DC 200	5	7	1.17
6 DC 300	7.33	6	2.36

** repeated in three sets

Supplementary Table. S4a: Representative segregation analysis of *PcINO1* introgressed selected transgenics under hygromycin in T_3 generation:

Plant Line	Total No. of seeds tested	Hygromycin sensitivity		Observed ratio	χ^2 - value	P-value
		Hyg^R seeds	Hyg^S seeds			
3PcINO1	30	23	7	3.28:1	0.044	0.833854
4PcINO1	45	33	12	2.75.:1	0.066	0.797252
7PcINO1	70	53	17	3.11:1	0.018	0.893273

**The result is significant at $p < 0.05$.

Supplementary Table. S4b: Relative comparison between germination efficiency of selected *PcINO1* introgressed homozygous transgenic lines under hygromycin selection in T_6 generation:

Plant Line	Total No. of seeds tested	Total no. of seeds germinated	Germination percentage (%)
3PcINO1	10	9	93.33
	10	10	
	10	9	
4PcINO1	10	10	96.66
	10	9	
	10	10	
7PcINO1	10	10	100**
	10	10	
	10	10	

Supplementary Table. S5a: Segregation analysis of *PcIMT1* introgressed transgenics in T_3 generation:

Plant Line	Total No. of seeds tested	Hygromycin sensitivity		Observed ratio	χ^2 - value	P-value
		Hyg^R seeds	Hyg^S seeds			
1APcIMT1	25	17	8	2.13:1	0.653	0.419042
10PcIMT1	38	29	9	3.22:1	0.035	0.851596
25PcIMT1	35	27	8	3.37:1	0.085	0.770633

**The result is significant at $p < 0.05$.

Supplementary Table. S5b: Relative comparison between germination efficiency of *PcIMT1* introgressed homozygous transgenic lines under hygromycin selection in T_4 generation:

Plant Line	Total No. of seeds tested	Total no. of seeds germinated	Germination percentage (%)
1APcIMT1	10	9	93.33
	10	10	
	10	9	
10PcIMT1	10	10	100**
	10	10	
	10	10	
25PcIMT1	10	8	90
	10	9	
	10	10	

Supplementary Table. S6a: Segregation analysis of *PcIMT1* introgressed transgenics in T_3 generation:

Plant Line	Total No. of seeds tested	Hygromycin sensitivity		Observed ration	χ^2 - value	P-value
		Hyg^R seeds	Hyg^S seeds			
4DC1	26	18	8	2.25:1	0.461	0.497157
5DC1	50	37	13	2.84:1	0.026	0.8719
6DC1	42	32	10	3.37:1	0.031	0.86024

**The result is significant at $p < 0.05$.

Supplementary Table. S6b: Relative comparison between germination efficiency of (*PcINO1+ PcIMT1*) introgressed homozygous transgenic lines (DC1) under hygromycin selection in T_4 generation:

Plant Line	Total No. of seeds tested	Total no. of seeds germinated	Germination percentage (%)
4DC1	10	9	93.33
	10	10	
	10	9	
5DC1	10	9	90
	10	9	
	10	9	
6DC1	10	10	100**
	10	10	
	10	10	

Supplementary Table. S7: GO terms for the 9 genes up-regulated in all experimental sets:

Locus ID	GO Term
LOC_Os05g19954	GO:0008152 metabolic process<br>GO:0006139 nucleobase, nucleoside, metabolic process<br>GO:0003824 catalytic activity<br>GO:0009536 plastid
LOC_Os03g03034	GO:0009058 biosynthetic process<br>GO:0009987 cellular process<br>GO:0008152 metabolic process<br>GO:0009607 response to biotic stimulus<br>GO:0019748 secondary metabolic process<br>GO:0003824 catalytic activity<br>GO:0005575 cellular component
LOC_Os09g28620	GO:0008152 metabolic process<br>GO:0016787 hydrolase activity<br>GO:0005575 cellular component
LOC_Os03g56260	GO:0009987 cellular process<br>GO:0008152 metabolic process<br>GO:0003774 motor activity<br>GO:0000166 nucleotide binding
LOC_Os02g37800	GO:0006629 lipid metabolic<br>GO:0016740 transferase activity<br>GO:0016020 membrane<br>GO:0005773 vacuole
LOC_Os01g61700	GO:0009056 catabolic process

	GO:0019538 protein metabolic process<br>GO:0003674 molecular function
LOC_Os02g46100	GO:0008150 biological process<br>GO:0005488 binding
LOC_Os08g01520	GO:0008152 metabolic process<br>GO:0005488 binding<br>GO:0003824 catalytic activity<br>GO:0003674 molecular function<br>GO:0019825 oxygen binding
LOC_Os01g71310	GO:0009058 biosynthetic process<br>GO:0009056 catabolic process<br>GO:0009987 cellular process<br>GO:0008152 metabolic process<br>GO:0007275 multicellular organismal development<br>GO:0006464 protein modification process<br>GO:0003824 catalytic activity<br>GO:0005773 vacuole

Supplementary Figure Legends

Figure S1. Sequence blast result of PcIMT1 protein with protein *AllergenOnline Database v15*

Figure S2. Sequence blast result of PcIMT1 protein with protein *AllergenOnline Database v15*

Figure S3. SEM study of IR64 calli at different developmental stages. (a) 10days old calli surface with minimal groovingl; (b) & (e) Protuberance from the outer surface of the calli showing (c) & (d) compact mass of cells but no proper embryo formation;(f) 20days old calli showing differentiating mass of cells protruding out from the outer layer of the calli with grooving; (h) uneven texture; (g) & (i) Bulging out of embryonic masses with different shapes; (j) Part for calli representing mass of torpedo shaped emdryogenic cells in (k); (l) Torpedo shaped embryogenic cells; (m) Globular shaped embryogenic cells;(n) 30 days old calli along with the growth of embryonic cells showing the presence of dead cells in the calla tissue; (o) Representative dead / sclerenchymatous tissues; (p) Presence of trichomes in the older calli tissue.

Figure S4. Improvised *Agrobacterium* mediated rice transformation protocol

Figure S5. Representative pictures explaining procedure of plant-transformation experiments and selection of presumptive transformants. (a), (b) and (c) *Agrobacterium*- infected IR-64 calli under hygromycin selection. (d) Regenerating plantlets from selected calli. (e) and (f) Putative transformants recovered from 2^{nd}round-hygromycin selection. PCR profiles of (g) and (h) *PcINO1* introgressed; (i) and (j) *PcIMT1* introgressed; (k), (l) and (m) DC1 (*PcINO1+PcIMT1*) introgressed putative T2 plant lines showing presence of *hptII* gene (1kb), *PcINO1* gene (1.5kb), *PcIMT1* gene (1.1kb) specific bands against specific primers in respective transformants lines.

Figure S6. Representative Southern blots exhibiting the presence of transgenes in T_2 transgenic lines. (a) The blot contained genomic DNA of *PcINO1* introgressed presumptive plants and has been charged with P^{32} labelled *PcINO1* part gene; (b) The blot contained genomic DNA of *PcIMT1* introgressed presumptive plants and has been charged with P^{32} labelled *PcIMT1* part gene; (c) The blot contained genomic DNA of DC1 (*PcINO1+PcIMT1*) introgressed presumptive plants and has been charged with P^{32} labelled *PcINO1* and (d)

PcIMT1 part genes in separate attempts. In all the cases, *Hind*III digested λDNA has been used as the marker.

Figure S7. Representative pictures showing generation and selection of *OsINO1* transformants followed by comparative salt-tolerant experiments with *PcINO1* lines based on phenotypic analysis under salt stress. (a) and (b) Representing PCR profiles of *OsINO1* overexpressing transformants exhibiting the presence of *hptII* gene. (c) Representative autoradiogram exhibiting Southern blot hybridization of different T_3 PCR positive *OsINO1* transformants probed with α-P^{32} labelled ~600bp *BamH*I digested DNA fragment from the coding region common to both *PcINOI/OsINO1* gene cloned in pCAMBIA-1301 vector. White arrows are indicative of transgene insertions. (d) and (e) Comparative study on effect of salt (NaCl) on Germination Percentage (GP %) of *PcINO1/OsINO1* transgenics on 10 DAG (Days After Germination) with control (rice IR64) grown on MS medium. (f) and (g) Comparative evaluation on effect of salt (NaCl; 0, 100, 200, 300 and 400 mM) on shoot and root length (cm) of *PcINO1/OsINO1* transgenics (T_3) and control (rice IR64) grown on MS medium for 15 days. (h) PIabs value of the transgenic lines (*PcINO1/OsINO1*) and control plant (IR64) in their vegetative and reproductive stages respectively. (i) Radar-plot presentation of photosynthetic parameters quantifying the behaviour of PSII in Rice transgenic plants (*PcINO1/OsINO1*) compared with the control (rice var.IR64). Biological samples are taken in triplicates. Data represented average of three replica sets ±SD ($P \leq 0.05$).

Figure S8. Schematic representation of major inositol dependent biochemical processes representing centralized connectivity through efflux and influx of inositol as the major compound into the network system of a plant.

Figure S9. The expression data of 8 unrelated and uncharacterized genes, generated in Genevisible.

Supplementary Table Legends

Table S1. TAIL-PCR analysis showing site of integration of particular transgenes in all transgenic lines.

Table S2. Accumulative list of primers used in different PCR experiments.

Table S3. Different parameters root morphology in transgenic plants and untransformed control lines under stressed and unstressed condition analyzed through DIRT software.

Table S4. (a & b) Segregation analysis of *PcINO1* introgressed transgenic lines in T_3 generation.

Table S5. (a & b) Segregation analysis of *PcIMT1* introgressed transgenic lines in T_3 generation.

Table S6. (a & b) Segregation analysis of (*PcINO1* + *PcIMT1*) introgressed DC1 transgenic lines in T_3 generation.

Table S7. GO terms for the 9 genes up-regulated in all experimental sets.
